# Lipid metabolism-related inflammatory indices and diabetic kidney disease in patients with type 2 diabetes mellitus: a cross-sectional study

**DOI:** 10.3389/fendo.2026.1934556

**Published:** 2026-10-13

**Authors:** Jing Liu, Xiaochan Tian, Mingjun Xu, Yao Xiao, Shuwu Wei, Xinyu Pan, Yue Shi, Hangyu Duan, Yu Zhang, Junping Wei

**Affiliations:** 1 Department of Endocrinology, Guang’anmen Hospital, China Academy of Chinese Medical Sciences, Beijing, China; 2 Clinical Medical College, Beijing University of Chinese Medicine, Beijing, China; 3 Department of Geriatrics, Affiliated Hospital of Shandong University of Traditional Chinese Medicine, Jinan, China; 4 Department of Endocrinology, Fuzhou Hospital of Traditional Chinese Medicine, Fuzhou, China; 5 Integrated Traditional Chinese and Western Medicine Diagnosis and Treatment Center, Chongqing General Hospital, Chongqing University, Chongqing, China; 6 Department of Nephrology, Xiyuan Hospital, China Academy of Chinese Medical Sciences, Beijing, China

**Keywords:** diabetic kidney disease, high-density lipoprotein cholesterol, lipid metabolism-related inflammatory indices, lymphocytes, monocytes, neutrophils, platelets, type 2 diabetes

## Abstract

**Background:**

Improving the prognosis of diabetic kidney disease (DKD) requires early diagnosis and treatment. This study examined the associations of lipid metabolism-related inflammatory indices (LMIIs)—the platelet-, neutrophil-, monocyte-, and lymphocyte-to-high-density lipoprotein cholesterol ratios (PHR, NHR, MHR, and LHR, respectively)—with DKD in patients with type 2 diabetes mellitus (T2DM).

**Methods:**

A total of 3,468 T2DM patients were enrolled in this cross-sectional study. Logistic regression models were employed to assess the association between LMIIs and DKD. Restricted cubic splines (RCS) were used to evaluate nonlinear relationships, and threshold effect analysis identified inflection points. Subgroup analyses and sensitivity analyses, including inverse probability of treatment weighting, were performed to test the robustness of the findings. Mediation analysis was conducted to explore indirect associations through selected markers. The candidate mediator was not included as a conventional covariate in the corresponding model. Receiver operating characteristic (ROC) curves were used to evaluate the discriminative performance of models incorporating the four LMIIs.

**Results:**

After multivariate adjustment, all four LMIIs showed significant positive associations with DKD (*P* for overall < 0.05). RCS analysis revealed substantial nonlinear dose - response relationships for PHR, NHR, and MHR (*P* for nonlinear = 0.009, 0.014, and 0.030, respectively), with inflection points at 2.65, 4.78, and 6.07, respectively. Subgroup analyses confirmed the robustness of these associations. However, lipid-lowering agent use significantly attenuated the NHR - DKD and MHR - DKD relationships (*P* for interaction = 0.032 and 0.007, respectively). Sensitivity analyses yielded consistent results. TG and ALB showed statistically significant indirect associations in exploratory mediation analyses. ROC analysis showed that the model incorporating NHR had a slightly higher area under the curve (AUC) than those incorporating PHR and LHR (*P* = 0.002 and 0.003, respectively).

**Conclusion:**

LMIIs were significantly associated with DKD and exhibited distinct nonlinear dose–response relationships with threshold effects. Lipid-lowering agent use attenuated the associations of NHR and MHR with DKD. NHR may provide modestly improved discrimination compared with PHR and LHR, while showing comparable performance to MHR. These findings highlight the potential relevance of lipid–inflammation interactions in DKD and warrant further prospective studies to clarify the underlying mechanisms and clinical implications.

## 1 Background

The global prevalence of diabetes has continued to increase. The International Diabetes Federation (IDF) estimates that there are 589 million adults with diabetes globally, and by 2050, that number is expected to rise to 853 million (1). One of the most prevalent microvascular complications of type 2 diabetes mellitus (T2DM) is diabetic kidney disease (DKD). Persistent proteinuria and a subsequent drop in the estimated glomerular filtration rate (eGFR) are the hallmarks of this condition. Additionally, it is the primary cause of end-stage kidney disease (ESKD), which increases mortality risk among patients with diabetes (2, 3). Globally, between 30% and 50% of individuals with diabetes will experience DKD (4). Therefore, early identification, risk stratification, and individualized management are crucial for preventing or delaying the onset and progression of DKD.

DKD is strongly associated with immune and inflammatory responses (5). Hyperglycemia-induced vasculitis is a key contributing factor. On the one hand, inflammatory signals activate platelets, leading to the expression of surface proteins and the release of granule contents, which then interact with leukocytes (e.g. neutrophils, monocytes, and lymphocytes). Moreover, substantial changes in blood cell counts can trigger a cascade of immune responses (6). On the other hand, inflammatory conditions upregulate the expression of adhesion molecules on endothelial cells, promoting leukocyte migration into the endothelium and inducing the release of pro-inflammatory mediators, thereby amplifying inflammatory signaling (7). Glomerular endothelial cells (GECs) are a vital part of the glomerular filtration barrier and are highly susceptible to inflammatory mediators, which can lead to functional impairment and facilitate the onset and advancement of DKD (8). One independent associated factor for DKD is platelet count (9). In patients with T2DM, elevated levels of the neutrophil-to-lymphocyte ratio (NLR), the platelet-to-lymphocyte ratio (PLR), and the systemic inflammatory index (SII, defined as platelet count × neutrophil count/lymphocyte count) are associated with DKD (10–12).

DKD is also associated with dyslipidemia. High-density lipoprotein cholesterol (HDL-C) is a protective lipid fraction with antioxidant and anti-inflammatory properties. HDL-C regulates immune cell function through multiple mechanisms, reduces intracellular oxidative stress, thereby attenuating inflammatory responses, and provides cardiovascular protection (13). Elevated HDL-C levels are associated with a reduced odds of DKD and renal function decline in patients with T2DM (14–16). Among patients with T2DM, the triglyceride/HDL-C ratio (TG/HDL-C) and the residual cholesterol to HDL-C ratio (RHR) demonstrate high predictive potential for DKD (17–20). However, other studies suggest that the predictive power of HDL-C for DKD in individuals with T2DM varies by sex and environmental factors (21). Under certain conditions, HDL-C may shift from an anti-inflammatory to a pro-inflammatory state (22). Therefore, reliance solely on HDL-C for identifying DKD may be insufficient.

Currently, composite inflammatory and lipid indices are emerging. Lipid metabolism-related inflammatory indices (LMIIs) are novel proxies of inflammatory reactions and metabolic dysfunction and are associated with various metabolic diseases, including PHR, NHR, MHR, and LHR. Previous studies have reported significant associations between LMIIs and peripheral artery disease (PAD), diabetic foot ulcers (DFU), cardiovascular-kidney-metabolic (CKM) syndrome, and metabolic dysfunction–associated steatohepatitis (MASLD) in patients with diabetes mellitus (23–27). Although previous studies have reported associations of NHR and MHR with DKD (28–30), the relationships of PHR and LHR with DKD, as well as the comparative associations of these four LMIIs in patients with diabetes, remain insufficiently understood. Investigating the relationship between LMII levels and DKD in diabetes may improve disease stratification and inform treatment strategies. Furthermore, LMIIs can be readily calculated from blood cell counts and HDL-C levels obtained in routine blood tests, making them a practical and accessible tool for disease assessment. In order to investigate the relationship between LMIIs and DKD, this study performed a cross-sectional analysis of T2DM individuals admitted to Guang’anmen Hospital’s Endocrinology Ward between 2017 and 2022.

## 2 Materials and methods

### 2.1 Participants and study design

To systematically collect comprehensive information on clinical characteristics, laboratory parameters, comorbidities and medication use, we included 4,149 patients with type 2 diabetes mellitus (T2DM) who were hospitalized at Guang’anmen Hospital between February 2017 and February 2022. The following criteria were used to exclude participants: (1) age < 20 years. (2) missing LMII-related variables, including neutrophils, monocytes, platelets, lymphocytes, or HDL-C, as well as eGFR and UACR. (3) acute diabetic complications, fractures, autoimmune disorders, cancer, acute inflammation or infection. (4) receipt of hemodialysis or peritoneal dialysis. 3,468 eligible individuals were included in the final analysis once these criteria were applied. This retrospective study was carried out in compliance with the Declaration of Helsinki and was authorized by the Medical Ethics Committee of Guang’anmen Hospital, China Academy of Chinese Medical Sciences (permission No. 2023-187-KY). The requirement for informed consent was waived by the Ethics Committee.

### 2.2 Diagnosis of T2DM and DKD

If any of the following conditions were satisfied, T2DM was diagnosed: (1) a discharge diagnosis of T2DM; (2) use of antidiabetic medications; (3) glycated hemoglobin (HbA1c) ≥ 6.5%; (4) fasting plasma glucose (FPG) ≥ 7.0 mmol/L after at least 8 hours of fasting, or random plasma glucose (RPG) ≥ 11.1 mmol/L.

DKD was defined as the presence of albuminuria (urinary albumin-to-creatinine ratio ≥30 mg/g, confirmed by repeated measurements within 3–6 months, with at least two of three measurements meeting this criterion) or impaired eGFR (<60 mL/min/1.73 m^2^, persisting for more than 3 months) (31). eGFR was calculated using the 2009 Chronic Kidney Disease Epidemiology Collaboration (CKD-EPI) creatinine equation and expressed as mL/min/1.73 m^2^. It should be noted that not all patients had repeated measurements of urine albumin-to-creatinine ratio (UACR) and eGFR within 3–6 months. In patients without repeat measurements, the diagnosis of DKD was reconfirmed after a comprehensive evaluation of their clinical history, laboratory findings, and diagnostic information documented at discharge.

### 2.3 Calculation of LMIIs

LMIIs, including PHR, NHR, MHR, and LHR, were calculated by dividing platelet, neutrophil, monocyte, and lymphocyte counts (×10^9^/L), respectively, by HDL-C (mmol/L). Given their different variability, MHR and PHR were rescaled to 10^8^/mmol and 10¹¹/mmol, respectively, whereas NHR and LHR remained in 10^9^/mmol.

### 2.4 Covariates

Stepwise regression analysis and clinical experience were used to choose the covariates. Patient admission data were taken from electronic health records, including sex, age, ethnicity, smoking and drinking use, diabetes duration, obesity status, history of hypertension, history of cardiovascular disease (CVD), medication use (antidiabetic medications, lipid-lowering agents, aspirin, and renin-angiotensin-aldosterone system inhibitors [RAAS inhibitors]), and baseline laboratory results obtained at admission. Data on multiple indicators were collected for subsequent analysis, including FPG, HbA1c, C-reactive protein (CRP), peripheral blood cell counts, hemoglobin (Hb), HDL-C, low-density lipoprotein cholesterol (LDL-C), triglycerides (TG), serum creatinine (Scr), eGFR, albumin (ALB), alanine aminotransferase (ALT), aspartate aminotransferase (AST), and UACR. Individually reported or physician-defined hypertension, antihypertensive drug use, systolic blood pressure (SBP) ≥140 mmHg, or diastolic blood pressure (DBP) ≥90 mmHg were used to identify hypertension. We referred to the guidelines for the diagnosis and treatment of Obesity in China (2024 edition) and defined obesity as body mass index (BMI) ≥28 kg/m^2^. Variables with a missingness rate exceeding 20% were excluded from the analysis, and missing values in the remaining variables were imputed using multiple imputation by chained equations (MICE).

### 2.5 Statistical analysis

To assess potential selection bias arising from the exclusion process, baseline demographic and clinical characteristics were compared between patients included in the final analysis and those excluded from the study. In the final analytical cohort, participants were classified according to the presence or absence of DKD. While categorical data were presented as counts and percentages (n,%), continuous variables were displayed as medians and interquartile ranges (median, IQR). Binary logistic regression was used to estimate odds ratios (ORs) and 95% confidence intervals (CIs) for the associations between LMIIs and DKD. Models were adjusted for demographic characteristics (sex, age, and ethnicity), laboratory markers, lifestyle factors (smoking and alcohol use), medical history (hypertension and CVD), and medication use.

**Supplementary Table S1** provides comprehensive information about missing data. Five imputed datasets were generated using MICE in R. The imputation model included DKD, LMIIs, sex, age, ethnicity, smoking and drinking use, diabetes duration, obesity, history of hypertension, history of CVD, medication use, and laboratory measurements, including FPG, HbA1c, CRP, Hb, LDL-C, TG, ALB, ALT, and AST. All variables with missing values were continuous and were imputed using predictive mean matching (PMM).

All analyses were performed separately in each imputed dataset, and parameter estimates and variances were pooled according to Rubin’s rules to derive pooled standard errors, 95% CIs, and P values. For RCS analyses, four-knot spline models were fitted in each dataset, with spline coefficients and covariance matrices pooled using Rubin’s rules to obtain pooled estimates of the overall and nonlinear associations. Prespecified subgroup and interaction analyses were similarly performed and pooled across datasets. Mediation analyses were conducted separately in each imputed dataset, with 1,000 bootstrap simulations used to estimate 95% confidence intervals. The results were pooled according to Rubin’s rules.

Restricted cubic splines (RCS) were applied to evaluate the nonlinear associations between LMIIs and DKD, and threshold effect models were used to identify inflection points. Subgroup analyses were performed stratified by age (<65, ≥65), sex (male, female), Han ethnicity (yes, no), hypertension (yes, no), CVD (yes, no), BMI (<24, 24-28, ≥28), Sodium-glucose cotransporter 2 inhibitors (SGLT2 inhibitor) (yes, no), Other antidiabetic medication (yes, no), lipid-lowering agents (yes, no), aspirin (yes, no), and RAAS inhibitors (yes, no). Sensitivity analyses were conducted using inverse probability of treatment weighting (IPTW), modeling BMI as a continuous variable instead of binary obesity status, and restricting the analysis to participants with repeated UACR and/or eGFR measurements to assess DKD chronicity. For the IPTW analysis, each LMII was dichotomized at its median value to define the lower and higher exposure groups. Propensity scores were estimated using binary logistic regression. Inverse probability weights were calculated based on the propensity scores and truncated at the 1st and 99th percentiles. Covariate balance was assessed using standardized mean differences (SMDs).

Exploratory mediation analyses were conducted to examine potential mediating effects. ROC curve analysis was used to assess model discrimination, with AUCs and their 95% confidence intervals estimated using the DeLong method. Pairwise comparisons of AUCs were performed using the DeLong test with Bonferroni adjustment for multiple comparisons. The incremental value of adding individual LMIIs to the base clinical model was assessed using net reclassification improvement (NRI) and integrated discrimination improvement (IDI).

This analysis was performed using R (v4.5.1). A two-sided *P* < 0.05 was considered statistically significant.

## 3 Results

### 3.1 Baseline characteristics

Baseline characteristics were broadly comparable between included and excluded patients, although some variables differed significantly, suggesting that the exclusion process was unlikely to have introduced substantial selection bias (**Supplementary Table S2**). A total of 3,468 participants with diabetes were included (**Figure 1**), of whom 2,485 (71.66%) were classified as non-DKD and 983 (28.34%) as DKD. As shown in **Table 1**, compared with non-DKD participants, the DKD group had substantially higher age, diabetes duration, FPG, HbA1c, CRP, PHR, NHR, MHR, LHR, Scr, UACR, TG, TC, and higher proportions of males, hypertension, and CVD (*P* < 0.05). In contrast, the DKD group had significantly reduced eGFR and ALB levels (*P* < 0.05). AST, LDL-C, ethnicity, smoking and alcohol use, obesity, antidiabetic medication use, lipid-lowering agents use, aspirin use, or RAAS inhibitors use did not, however, differ significantly between the groups (*P* > 0.05).

**Figure 1:**
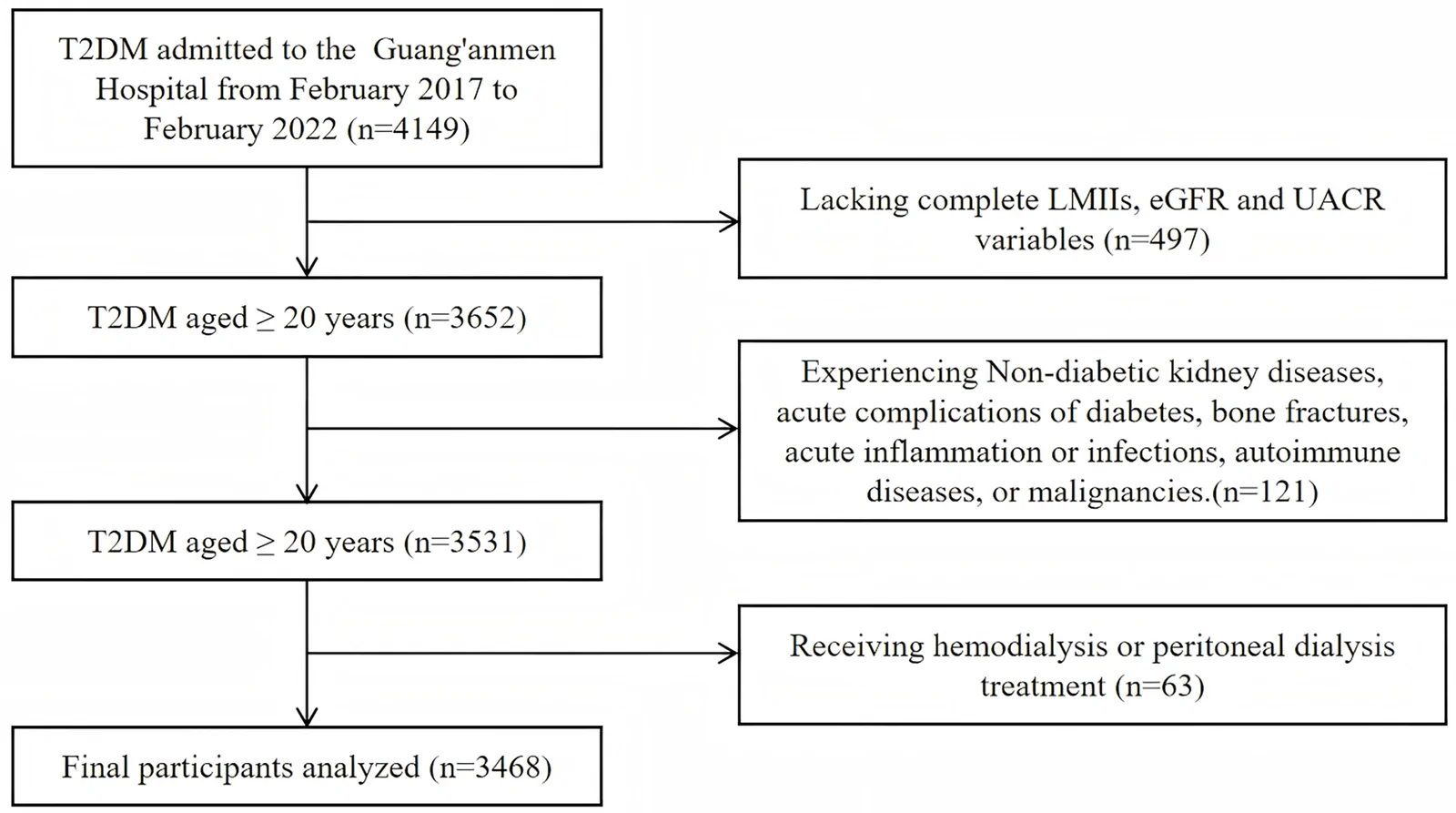
Flowchart of patient selection.

**Table 1:** Baseline characteristics of the study population.

Characteristic	Non-DKD	DKD	*P* value
N	2485	983	
Age (years)	59.00 (51.00, 66.00)	63.00 (54.00, 69.00)	<0.001
Duration of diabetes (years)	11.00 (5.00, 17.00)	12.00 (6.00, 18.25)	0.001
FPG (mmol/L)	10.32 (7.90, 13.91)	11.30 (8.21, 15.46)	<0.001
HbA1c (%)	8.40 (7.20, 9.80)	8.70 (7.50, 10.00)	<0.001
CRP (mg/dL)	1.00 (0.25, 2.05)	1.00 (0.25, 3.13)	<0.001
PHR (10^11^/mmol)	1.98 (1.56, 2.47)	2.07 (1.73, 2.61)	<0.001
NHR (10^9^/mmol)	3.54 (2.69, 4.68)	4.19 (3.17, 5.45)	<0.001
MHR (10^8^/mmol)	3.01 (2.30, 3.96)	3.46 (2.68, 4.51)	<0.001
LHR (10^9^/mmol)	1.51 (1.12, 1.98)	1.57 (1.17, 2.03)	0.039
Hb (g/L)	142.00 (132.00, 152.00)	137.00 (124.00, 150.00)	<0.001
Scr (umol/L)	59.00 (51.00, 69.10)	70.60 (56.90, 94.00)	<0.001
eGFR (mL/min per 1.73 m^2^)	103.40 (96.40, 111.10)	95.10 (69.40, 105.65)	<0.001
UACR (mg/g)	10.92 (6.14, 15.00)	135.86 (53.00, 407.85)	<0.001
ALB (g/L)	44.00 (41.80, 46.21)	42.20 (39.60, 44.70)	<0.001
ALT (U/L)	20.00 (14.70, 29.00)	18.40 (13.25, 26.65)	<0.001
AST (U/L)	19.70 (16.00, 25.00)	19.00 (15.80, 24.25)	0.047
LDL-C (mmol/L)	2.94 (2.37, 3.53)	2.97 (2.34, 3.67)	0.212
TG (mmol/L)	1.45 (1.05, 2.10)	1.74 (1.24, 2.54)	<0.001
TC (mmol/L)	4.62 (3.85, 5.39)	4.66 (3.83, 5.66)	0.047
Sex (n, %)			0.012
Male	1271 (51.15%)	549 (55.85%)	
Female	1214 (48.85%)	434 (44.15%)	
Ethnicity (n, %)			0.940
Han ethnicity	2324 (93.52%)	920 (93.59%)	
Others	161 (6.48%)	63 (6.41%)	
Drinking (n, %)			1.000
No	1537 (61.85%)	608 (61.85%)	
Yes	948 (38.15%)	375 (38.15%)	
Smoking (n, %)			0.144
No	1543 (62.09%)	584 (59.41%)	
Yes	942 (37.91%)	399 (40.59%)	
Hypertension (n, %)			<0.001
No	1149 (46.24%)	190 (19.33%)	
Yes	1336 (53.76%)	793 (80.67%)	
CVD (n, %)			<0.001
No	1879 (75.61%)	640 (65.11%)	
Yes	606 (24.39%)	343 (34.89%)	
Obesity (n, %)			0.193
No	2265 (91.15%)	882 (89.73%)	
Yes	220 (8.85%)	101 (10.27%)	
Antidiabetic medications (n, %)			0.563
No	186 (7.48%)	68 (6.92%)	
Yes	2299 (92.52%)	915 (93.08%)	
Lipid-lowering agents (n, %)			0.773
No	781 (31.43%)	304 (30.93%)	
Yes	1704 (68.57%)	679 (69.07%)	
Aspirin (n, %)			0.106
No	1627 (65.47%)	615 (62.56%)	
Yes	858 (34.53%)	368 (37.44%)	
RAAS inhibitors (n, %)			0.196
No	1665 (67.00%)	636 (64.70%)	
Yes	820 (33.00%)	347 (35.30%)	

FPG, Fasting Plasma Glucose; HbA1c, Glycosylated hemoglobin.; CRP, C-reactive protein; PHR, Platelet-to-high-density lipoprotein cholesterol ratio; NHR, neutrophil-to-high-density lipoprotein cholesterol ratio; MHR, monocyte-to-high-density lipoprotein cholesterol ratio; LHR, lymphocyte-to-high-density lipoprotein cholesterol ratio; Hb, Hemoglobin; Scr, Serum creatinine; eGFR, estimated glomerular filtration rate; UACR, urine albumin-to-creatinine ratio; ALB, Albumin; ALT, Alanine Aminotransferase; AST, Aspartate Aminotransferase; LDL-C, Low-Density Lipoprotein Cholesterol; TG, Triglycerides; TC, Total Cholesterol; CVD, Cardiovascular Disease; RAAS inhibitors, Renin-Angiotensin-Aldosterone System inhibitors.

### 3.2 Logistic regression analysis

To evaluate the independent association between LMIIs and DKD, four stepwise logistic regression models were built. LMIIs were categorized into quartiles, with the reference group being the lowest quartile. Model 1 included only LMIIs as the independent variable. Model 2 adjusted for demographic and disease duration factors (gender, age, ethnicity, and diabetes duration) to control for key confounders. Model 3 further adjusted for relevant variables (FPG, HbA1c, CRP, Hb, ALB, ALT, AST, LDL-C, TG, hypertension, CVD, obesity, antidiabetic medications, lipid-lowering agents, aspirin, RAAS inhibitors, smoking and drinking). Additional analyses evaluating each individual component (neutrophil count, monocyte count, lymphocyte count, platelet count, and HDL-C) as an independent exposure showed their respective associations with the outcome and are presented alongside the corresponding ratio-based models in **Supplementary Table S3**.

As shown in **Table 2**, all indices showed a substantial positive association with DKD when LMIIs were examined as continuous variables. For every increase in NHR (10^9^mmol), MHR (10^8^mmol), PHR (10^11^mmol), and LHR (10^9^mmol), the odds ratios (ORs) for DKD in the fully adjusted, unweighted model were ORs of 1.20 (95% CI: 1.07–1.34), 1.20 (95% CI: 1.14–1.26), 1.18 (95% CI: 1.11–1.26), and 1.21 (95% CI: 1.07–1.37), respectively. Results of trend tests indicated that all four indices exhibited a significant dose-response relationship from Q1 to Q4 (*P* for trend < 0.001), indicating that higher LMII levels were associated with higher odds of prevalent DKD.

**Table 2:** Associations of LMIIs with DKD.

Categories	Model 1 OR(95% CI)	*P-*value	Model 2 OR(95% CI)	*P-*value	Model 3 OR(95% CI)	*P-*value
PHR	1.22 (1.11, 1.34)	<0.001	1.33 (1.20, 1.46)	<0.001	1.20 (1.07, 1.34)	0.001
PHR (category)						
Q1 (≤1.59)	Ref.		Ref.		Ref.	
Q2 (1.59, 2.01]	1.35 (1.08, 1.67)	0.007	1.39 (1.11, 1.73)	0.004	1.38 (1.09-1.75)	0.008
Q3 (2.01, 2.51]	1.38 (1.12, 1.72)	0.003	1.50(1.21, 1.87)	<0.001	1.50 (1.18-1.91)	<0.001
Q4 (>2.51)	1.66 (1.35, 2.06)	<0.001	1.93 (1.55, 2.41)	<0.001	1.66 (1.29-2.12)	<0.001
*P* for trend		<0.001		<0.001		<0.001
NHR	1.22 (1.17, 1.27)	<0.001	1.22 (1.17, 1.27)	<0.001	1.20 (1.14, 1.26)	<0.001
NHR (category)						
Q1 (≤2.79)	Ref.		Ref.		Ref.	
Q2 (2.79, 3.72]	1.30 (1.04, 1.64)	0.025	1.25 (0.99, 1.58)	0.059	1.21 (0.94, 1.55)	0.144
Q3 (3.72, 4.93]	1.96 (1.58, 2.45)	<0.001	1.92 (1.54, 2.41)	<0.001	1.82 (1.42, 2.33)	<0.001
Q4 (>4.93)	2.70 (2.18, 3.36)	<0.001	2.63 (2.11, 3.28)	<0.001	2.35 (1.82, 3.03)	<0.001
P for trend		<0.001		<0.001		<0.001
MHR	1.21 (1.16, 1.27)	<0.001	1.21 (1.15, 1.27)	<0.001	1.18 (1.11, 1.26)	<0.001
MHR (category)						
Q1 (≤2.38)	Ref.		Ref.		Ref.	
Q2 (2.38, 3.15]	1.29 (1.03, 1.62)	0.026	1.28 (1.02, 1.61)	0.036	1.18 (0.92, 1.51)	0.200
Q3 (3.15, 4.10]	1.86 (1.50, 2.32)	<0.001	1.79 (1.43, 2.24)	<0.001	1.64 (1.28, 2.11)	<0.001
Q4 (>4.10)	2.28 (1.84, 2.83)	<0.001	2.25 (1.79, 2.83)	<0.001	2.00 (1.54, 2.60)	<0.001
P for trend		<0.001		<0.001		<0.001
LHR	1.09 (0.99, 1.20)	0.089	1.18 (1.07, 1.31)	0.002	1.21 (1.07, 1.37)	0.003
LHR (category)						
Q1 (≤1.13)	Ref.		Ref.		Ref.	
Q2 (1.13, 1.52]	1.08 (0.87, 1.34)	0.481	1.12 (0.90, 1.38)	0.319	1.11 (0.88, 1.41)	0.387
Q3 (1.52, 2.00]	1.20 (0.98, 1.48)	0.085	1.31 (1.06, 1.63)	0.013	1.43 (1.13, 1.82)	0.003
Q4 (>2.00)	1.21 (0.98, 1.50)	0.071	1.40 (1.13, 1.74)	0.002	1.49 (1.16, 1.92)	0.002
P for trend		0.042		<0.001		<0.001

Model 1 was unadjusted.

Model 2 was adjusted for age, sex, ethnicity, and diabetes duration.

Model 3 was adjusted for age, sex, ethnicity, diabetes duration, FPG, HbA1c, CRP, Hb, ALB, ALT, AST, LDL-C, TG, hypertension, CVD, obesity, antidiabetic medications, lipid-lowering agents, aspirin, RAAS inhibitors, smoking and drinking.

LMIIs, lipid metabolism-related inflammatory indices; PHR, platelet-to-high-density lipoprotein cholesterol ratio; NHR, neutrophil-to-high-density lipoprotein cholesterol ratio; MHR, monocyte-to-high-density lipoprotein cholesterol ratio; LHR, lymphocyte-to-high-density lipoprotein cholesterol ratio; DKD, diabetic kidney disease; FPG, Fasting Plasma Glucose; HbA1c, Glycosylated hemoglobin.; CRP, C-reactive protein; Hb, Hemoglobin; ALB, Albumin; ALT, Alanine Aminotransferase; AST, Aspartate Aminotransferase; LDL-C, Low-Density Lipoprotein Cholesterol; TG, Triglycerides; CVD, Cardiovascular Disease; RAAS inhibitors, Renin-Angiotensin-Aldosterone System inhibitors. 95% CI, 95% confidence interval; OR, odds ratio.

### 3.3 RCS curves and threshold analysis

RCS analysis was performed to evaluate the dose–response association between LMIIs and DKD. The RCS model uses four knots, which are located at P5, P35, P65, and P95 based on pre-selected percentiles. According to **Figure 2**, after adjusting for relevant confounders (sex, age, ethnicity, smoking and drinking use, diabetes duration, laboratory markers, obesity status, history of hypertension, history of CVD, medication use), the overall associations between LMIIs and DKD were statistically notable (*P* for overall < 0.01), and notable nonlinear relationships were observed for PHR (*P* for nonlinear = 0.009), NHR (*P* for nonlinear = 0.014), and MHR (*P* for nonlinear = 0.03). As shown in **Tables 3**–**6**, the log-likelihood ratio tests indicated that the two-piecewise models provided a significantly better fit than the standard linear models for PHR (*P* for LRT <0.001), NHR (*P* for LRT <0.001), MHR (*P* for LRT <0.001), and LHR (*P* for LRT = 0.003). Threshold effect analyses identified inflection points of 2.65 (95% CI: 2.07 - 3.22) for PHR, 4.78 (95% CI: 3.50 - 6.07) for NHR, 6.07 (95% CI: 4.45 - 7.69) for MHR, and 1.64 (95% CI: 0.93 - 2.36) for LHR. Below these thresholds, each one-unit increase in PHR, NHR, MHR, and LHR was associated with a 50% (OR = 1.50, 95% CI: 1.21 - 1.85, *P* < 0.001), 36% (OR = 1.36, 95% CI: 1.21 - 1.53, *P* < 0.001), 27% (OR = 1.27, 95% CI: 1.17 - 1.38, *P* < 0.001), and 54% (OR = 1.54, 95% CI: 1.06 - 2.25, *P* = 0.023) higher odds of prevalent DKD, respectively. Above the inflection points, none of these indices was significantly associated with DKD (PHR: *P* = 0.374; NHR: *P* = 0.051; MHR: *P* = 0.509; LHR: *P* = 0.523).

**Figure 2:**
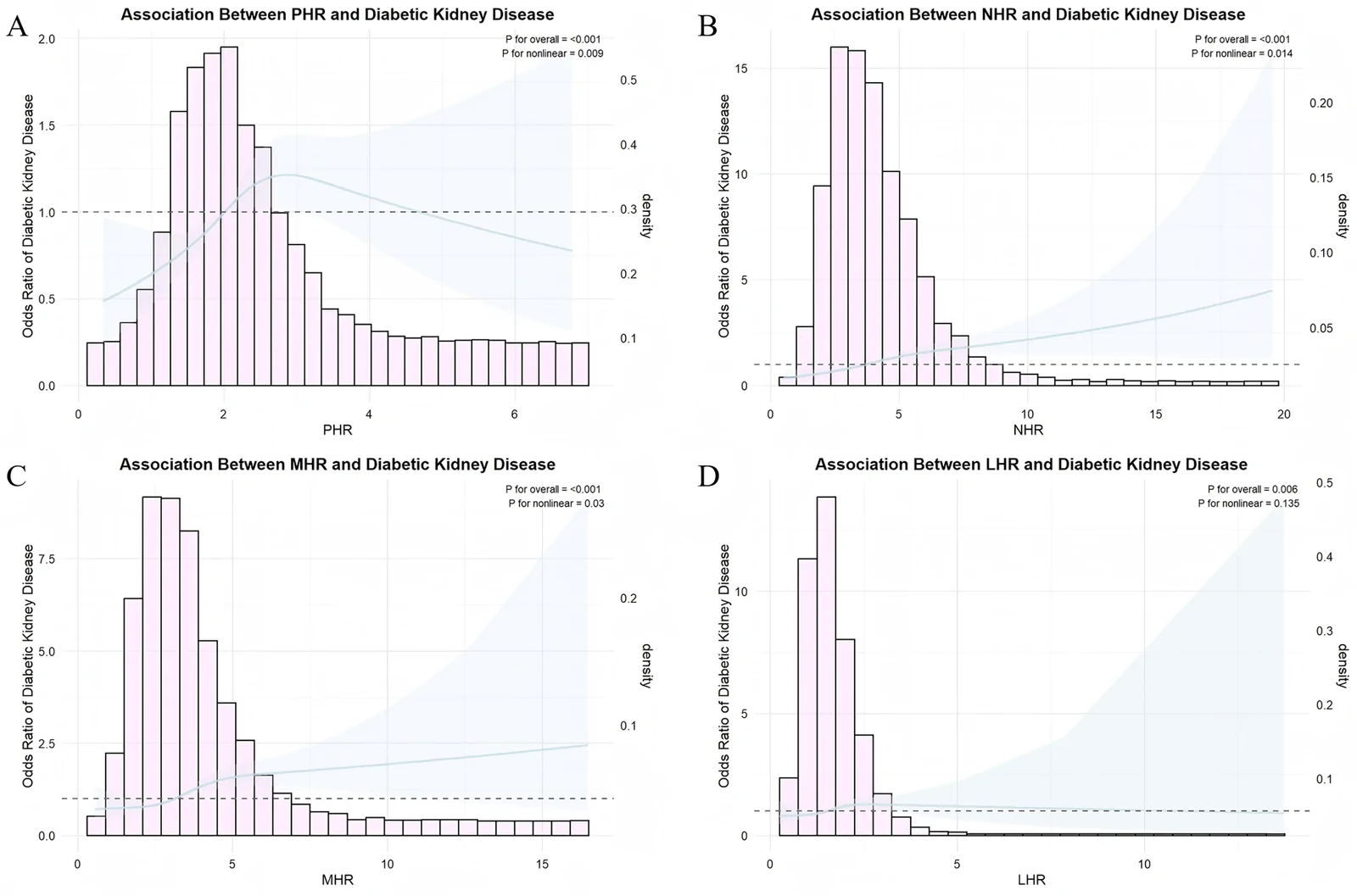
Associations between lipid metabolism-related inflammatory indices (LMIIs) and diabetic kidney disease (DKD) using restricted cubic spline (RCS) plots. **(A)** Association between platelet-to-high-density lipoprotein cholesterol ratio (PHR) and DKD; **(B)** association between neutrophil-to-high-density lipoprotein cholesterol ratio (NHR) and DKD; **(C)** association between monocyte-to-high-density lipoprotein cholesterol ratio (MHR) and DKD; and **(D)** association between lymphocyte-to-high-density lipoprotein cholesterol ratio (LHR) and DKD.

**Table 3:** Threshold effect analysis of the association between PHR and DKD.

Model	Variable	Value (95% CI)	Adjusted OR (95% CI)	*P*-value
Fitting by standard linear model	PHR		1.20 (1.07, 1.34)	0.001
Fitting by two-piecewise linear model				
	Inflection point	2.65 (2.07 - 3.22)		
	PHR < threshold		1.50 (1.21, 1.85)	<0.001
	PHR > threshold		0.89 (0.68, 1.15)	0.374
	Log likelihood ratio			<0.001

Age, sex, ethnicity, and diabetes duration, FPG, HbA1c, CRP, Hb, ALB, ALT, AST, LDL-C, TG, hypertension, CVD, obesity, antidiabetic medications, lipid-lowering agents, aspirin, RAAS inhibitors, smoking and drinking were adjusted.

PHR, Platelet-to-high-density lipoprotein cholesterol ratio; DKD, diabetic kidney disease; FPG, Fasting Plasma Glucose; HbA1c, Glycosylated hemoglobin.; CRP, C-reactive protein; Hb, Hemoglobin; ALB, Albumin; ALT, Alanine Aminotransferase; AST, Aspartate Aminotransferase; LDL-C, Low-Density Lipoprotein Cholesterol; TG, Triglycerides; CVD, Cardiovascular Disease; RAAS inhibitors, Renin-Angiotensin-Aldosterone System inhibitors. 95% CI, 95% confidence interval; OR, odds ratio.

**Table 4:** Threshold effect analysis of the association between NHR and DKD.

Model	Variable	Value (95% CI)	Adjusted OR (95% CI)	*P*-value
Fitting by standard linear model	NHR		1.20 (1.14, 1.26)	<0.001
Fitting by two-piecewise linear model				
	Inflection point	4.78 (3.50 - 6.07)		
	NHR < threshold		1.36 (1.21, 1.53)	<0.001
	NHR > threshold		1.09 (1.00, 1.20)	0.051
	Log likelihood ratio			<0.001

Age, sex, ethnicity, and diabetes duration, FPG, HbA1c, CRP, Hb, ALB, ALT, AST, LDL-C, TG, hypertension, CVD, obesity, antidiabetic medications, lipid-lowering agents, aspirin, RAAS inhibitors, smoking and drinking were adjusted.

NHR, neutrophil-to-high-density lipoprotein cholesterol ratio; DKD, diabetic kidney disease; FPG, Fasting Plasma Glucose; HbA1c, Glycosylated hemoglobin.; CRP, C-reactive protein; Hb, Hemoglobin; ALB, Albumin; ALT, Alanine Aminotransferase; AST, Aspartate Aminotransferase; LDL-C, Low-Density Lipoprotein Cholesterol; TG, Triglycerides; CVD, Cardiovascular Disease; RAAS inhibitors, Renin-Angiotensin-Aldosterone System inhibitors. 95% CI, 95% confidence interval; OR, odds ratio.

**Table 5:** Threshold effect analysis of the association between MHR and DKD.

Model	Variable	Value (95% CI)	Adjusted OR (95% CI)	*P*-value
Fitting by standard linear model	MHR		1.18 (1.11, 1.26)	<0.001
Fitting by two-piecewise linear model				
	Inflection point	6.07 (4.45 - 7.69)		
	MHR < threshold		1.27 (1.17, 1.38)	<0.001
	MHR > threshold		0.92 (0.73, 1.17)	0.509
	Log likelihood ratio			<0.001

Age, sex, ethnicity, and diabetes duration, FPG, HbA1c, CRP, Hb, ALB, ALT, AST, LDL-C, TG, hypertension, CVD, obesity, antidiabetic medications, lipid-lowering agents, aspirin, RAAS inhibitors, smoking and drinking were adjusted.

MHR, monocyte-to-high-density lipoprotein cholesterol ratio; DKD, diabetic kidney disease; FPG, Fasting Plasma Glucose; HbA1c, Glycosylated hemoglobin.; CRP, C-reactive protein; Hb, Hemoglobin; ALB, Albumin; ALT, Alanine Aminotransferase; AST, Aspartate Aminotransferase; LDL-C, Low-Density Lipoprotein Cholesterol; TG, Triglycerides; CVD, Cardiovascular Disease; RAAS inhibitors, Renin-Angiotensin-Aldosterone System inhibitors. 95% CI, 95% confidence interval; OR, odds ratio.

**Table 6:** Threshold effect analysis of the association between LHR and DKD.

Model	Variable	Value (95% CI)	Adjusted OR (95% CI)	*P*-value
Fitting by standard linear model	LHR		1.21 (1.07, 1.37)	0.003
Fitting by two-piecewise linear model				
	Inflection point	1.64 (0.93 - 2.36)		
	LHR < threshold		1.54 (1.06, 2.25)	0.023
	LHR > threshold		1.07 (0.87, 1.31)	0.523
	Log likelihood ratio			0.003

Age, sex, ethnicity, and diabetes duration, FPG, HbA1c, CRP, Hb, ALB, ALT, AST, LDL-C, TG, hypertension, CVD, obesity, antidiabetic medications, lipid-lowering agents, aspirin, RAASi nhibitors, smoking and drinking were adjusted.

LHR, lymphocyte-to-high-density lipoprotein cholesterol ratio; DKD, diabetic kidney disease; FPG, Fasting Plasma Glucose; HbA1c, Glycosylated hemoglobin.; CRP, C-reactive protein; Hb, Hemoglobin; ALB, Albumin; ALT, Alanine Aminotransferase; AST, Aspartate Aminotransferase; LDL-C, Low-Density Lipoprotein Cholesterol; TG, Triglycerides; CVD, Cardiovascular Disease; RAAS inhibitors, Renin-Angiotensin-Aldosterone System inhibitors. 95% CI, 95% confidence interval; OR, odds ratio.

### 3.4 Subgroup analysis

To further assess the stability of the association between LMIIs and DKD and explore potential heterogeneity across populations, subgroup and interaction analyses were performed. As shown in **Figure 3**, subgroup analyses had little impact on the key association between LMIIs and DKD, indicating that the overall findings were robust; however, significant effect modification was observed in certain subgroups. In subgroup analyses stratified by lipid-lowering agent use, significant interactions were observed for NHR and MHR (*P* for interaction = 0.032 and 0.007, respectively), with stronger associations with DKD observed among patients not receiving lipid-lowering agents. No significant interactions were observed for PHR or LHR (*P* for interaction = 0.182 and 0.382, respectively).

**Figure 3:**
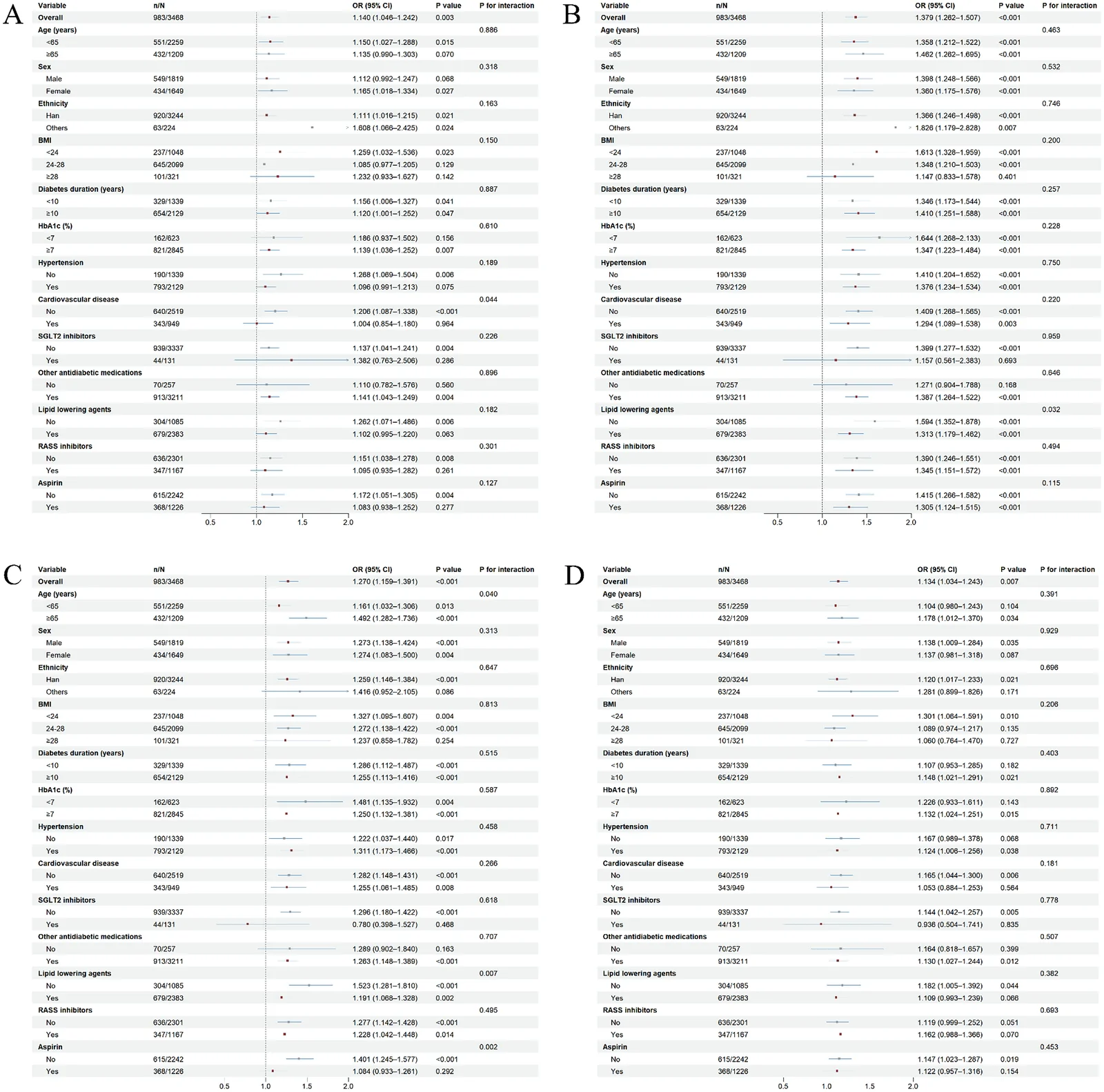
Subgroup analyses of the associations between lipid metabolism-related inflammatory indices (LMIIs) and diabetic kidney disease (DKD). **(A)** Association between platelet-to-high-density lipoprotein cholesterol ratio (PHR) and DKD; **(B)** association between neutrophil-to-high-density lipoprotein cholesterol ratio (NHR) and DKD; **(C)** association between monocyte-to-high-density lipoprotein cholesterol ratio (MHR) and DKD; and **(D)** association between lymphocyte-to-high-density lipoprotein cholesterol ratio (LHR) and DKD.

### 3.5 Sensitivity analysis

Sensitivity analyses yielded results broadly consistent with those of the primary analyses. In the IPTW analysis, higher PHR, NHR, MHR, and LHR were significantly associated with increased odds of DKD, with ORs of 1.35 (95% CI: 1.13–1.60), 1.86 (95% CI: 1.56–2.22), 1.61 (95% CI: 1.34–1.93), and 1.35 (95% CI: 1.14–1.61), respectively (all *P* < 0.001) (**Supplementary Table S4**). The distributions of the IPTW weights and the standardized mean differences (SMDs) before and after weighting are presented in **Supplementary Tables S5** and **S6**.

When BMI was modeled as a continuous variable instead of using binary obesity status, the associations remained largely unchanged (**Supplementary Table S7**). All four LMIIs showed significant positive trends across quartiles (all *P* for trend < 0.01). Compared with the lowest quartile, the highest quartile was associated with higher odds of prevalent DKD for PHR (OR = 1.56, 95% CI: 1.22–2.00), NHR (OR = 2.22, 95% CI: 1.72–2.86), MHR (OR = 1.87, 95% CI: 1.43–2.43), and LHR (OR = 1.42, 95% CI: 1.11–1.83). These findings were consistent with those of the primary analyses, further supporting the robustness of the observed associations.

Among the 3,468 patients, 1,962 had repeated UACR and/or eGFR measurements and were included in the repeated-measurement sensitivity analysis. Of these, 566 met the prespecified chronicity criteria for DKD, while the remaining 1,396 did not and were classified as non-DKD. The positive associations between LMIIs and DKD remained consistent with those in the primary analysis (**Supplementary Table S8**).

### 3.6 Mediation analysis

Exploratory mediation analyses were conducted to evaluate potential indirect associations between LMIIs and DKD, with selected biomarkers considered as potential mediators (**Supplementary Tables S9**, **S10**). As shown in **Figure 4**, TG and ALB showed statistically significant indirect associations. TG showed significant indirect associations for all four LMIIs, with proportions mediated ranging from 12.26% to 59.42%. ALB showed significant indirect associations for three LMIIs, excluding LHR, with proportions mediated ranging from 12.44% to 19.58%.

**Figure 4:**
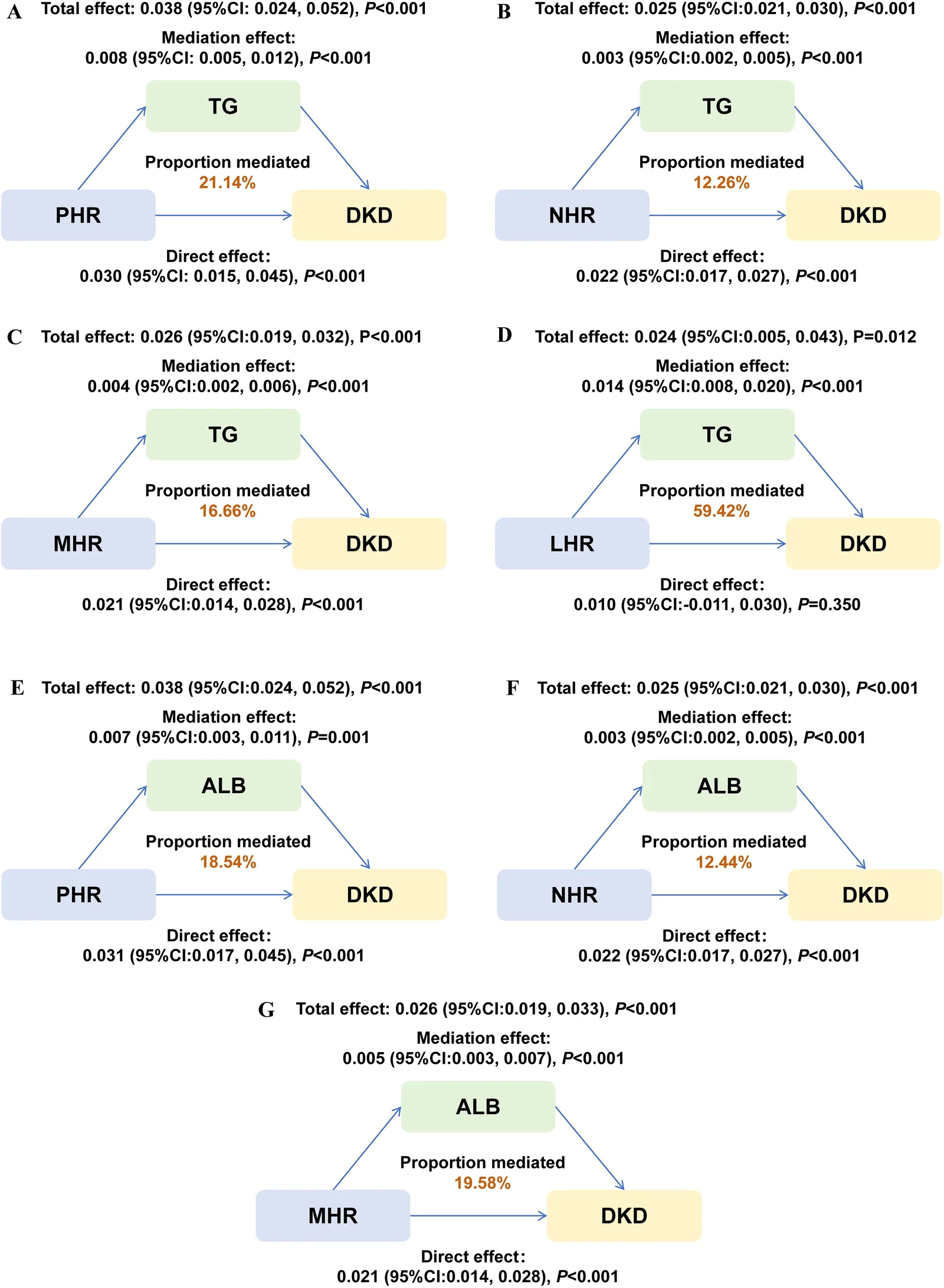
The mediating roles of triglyceride (TG) and albumin (ALB) in the associations between lipid metabolism-related inflammatory indices (LMIIs) and diabetic kidney disease (DKD). **(A–D)** TG as a mediator in the associations of platelet-to-high-density lipoprotein cholesterol ratio (PHR), neutrophil-to-high-density lipoprotein cholesterol ratio (NHR), monocyte-to-high-density lipoprotein cholesterol ratio (MHR), and lymphocyte-to-high-density lipoprotein cholesterol ratio (LHR) with DKD, respectively; **(E–G)** ALB as a mediator in the associations of PHR, NHR, and MHR with DKD, respectively.

### 3.7 ROC analysis

We conducted ROC curve analysis to evaluate the discriminative ability of LMIIs for DKD (**Figure 5**). As shown in **Table 7**, the AUCs of the multivariable models incorporating PHR, NHR, MHR, and LHR were 0.750 (95% CI, 0.732 - 0.768), 0.759 (95% CI, 0.741 - 0.776), 0.754 (95% CI, 0.736 - 0.772) and 0.750 (95% CI, 0.731 - 0.768). Overall, the LMIIs showed moderate discriminative ability for DKD. ROC analysis demonstrated that NHR had a significantly higher AUC than PHR (*P* = 0.002) and LHR (*P* = 0.003) after Bonferroni adjustment. However, no statistically significant differences were observed between NHR and MHR (*P* = 0.220). Further details are provided in **Supplementary Table S11.**

**Figure 5:**
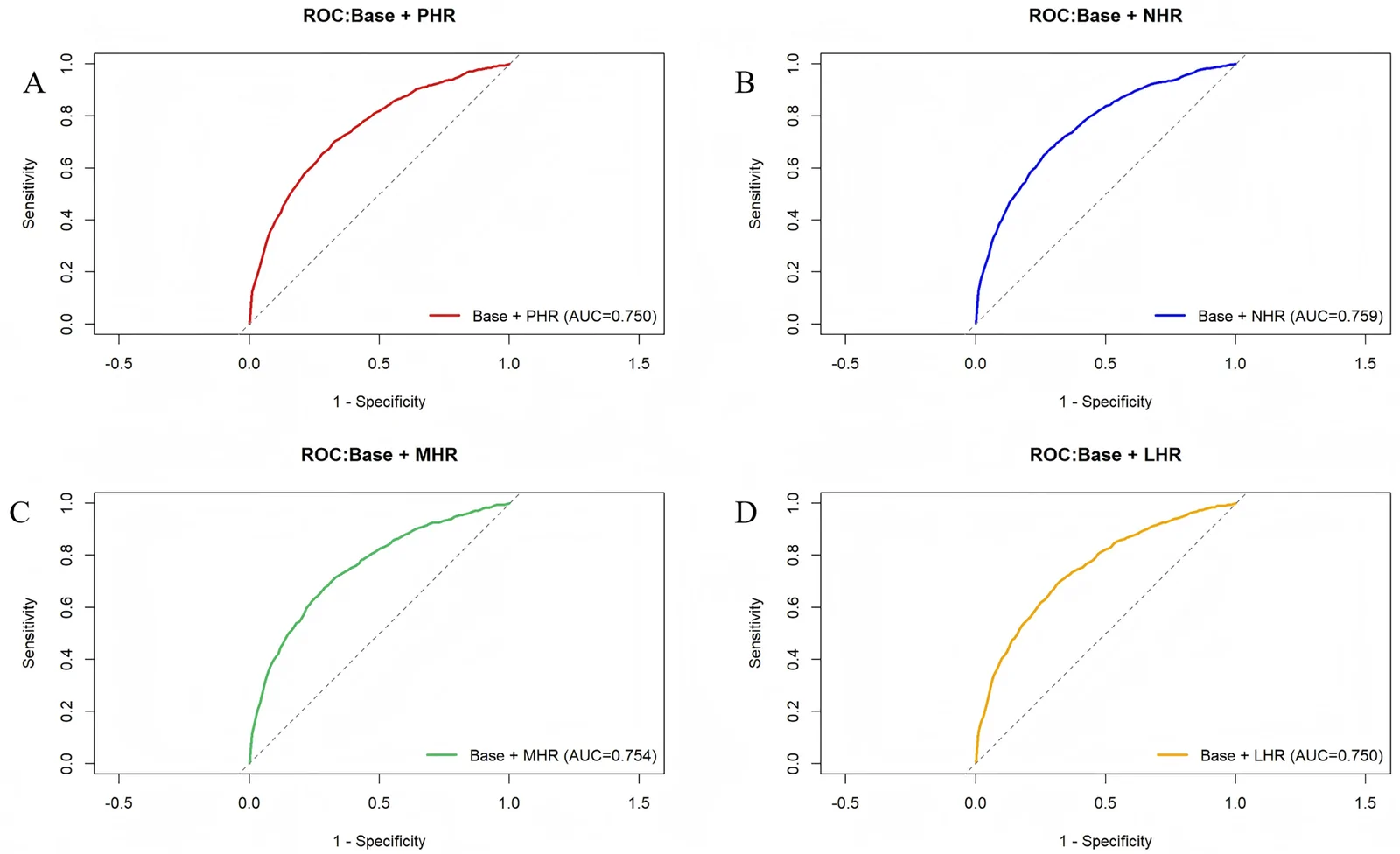
Receiver operating characteristic (ROC) curves for diabetic kidney disease (DKD). **(A)** Platelet-to-high-density lipoprotein cholesterol ratio (PHR); **(B)** neutrophil-to-high-density lipoprotein cholesterol ratio (NHR); **(C)** monocyte-to-high-density lipoprotein cholesterol ratio (MHR); and **(D)** lymphocyte-to-high-density lipoprotein cholesterol ratio (LHR).

**Table 7:** Comparison of AUC values between LMIIs.

Test	AUC	Unadjusted *P*	Bonferroni-adjusted *P*	95% CI	Optimal threshold	Specificity	Sensitivity
Base + PHR	0.750	0.00041	0.00246	0.732-0.768	0.284	0.689	0.689
Base +NHR	0.759	NA	NA	0.741-0.776	0.302	0.729	0.661
Base +MHR	0.754	0.03667	0.22001	0.736-0.772	0.301	0.727	0.661
Base +LHR	0.750	0.00053	0.00320	0.731-0.768	0.282	0.686	0.694

The threshold represents the optimal predicted probability from the corresponding multivariable logistic regression model and is not a cutoff value of the LMII itself. P values were calculated for pairwise comparisons of AUCs between each LMII-based model and the NHR-based model. Bonferroni-adjusted P values were used to account for multiple comparisons. The base model includes age, sex, ethnicity, diabetes duration, FPG, HbA1c, CRP, Hb, ALB, ALT, AST, LDL-C, TG, hypertension, CVD, obesity, antidiabetic medications, lipid-lowering agents, aspirin, RAAS inhibitors, smoking and drinking.

AUC, area under the curve; LMIIs, lipid metabolism-related inflammatory indices; PHR, Platelet-to-high-density lipoprotein cholesterol ratio; NHR, neutrophil-to-high-density lipoprotein cholesterol ratio; MHR, monocyte-to-high-density lipoprotein cholesterol ratio; LHR, lymphocyte-to-high-density lipoprotein cholesterol ratio.

### 3.8 Incremental discrimination and reclassification of LMIIs for prevalent DKD

Adding individual LMIIs to the core clinical model resulted in significant improvements in case reclassification and integrated discrimination, although the increases in AUC were generally modest (**Supplementary Table S12**). Using the continuous NRI approach, NHR showed the greatest incremental improvement among the individual indices, with an NRI of 0.276 and an IDI of 0.014 (both *P* < 0.001), followed by MHR (NRI, 0.176; IDI, 0.009; both *P* < 0.001). PHR and LHR also significantly improved reclassification and integrated discrimination, although their gains in AUC were limited.

## 4 Discussion

In this research, the associations between the four LMIIs and the odds of DKD among T2DM patients were assessed, and the potential mediating roles of relevant indicators were further investigated. Overall, higher levels of PHR, NHR, MHR, and LHR were positively associated with the odds of prevalent DKD. LMIIs may provide additional information for case reclassification and integrated discrimination beyond conventional clinical factors. However, this incremental value was statistically detectable but modest in magnitude. In our exploratory mediation analysis, TG and ALB were identified as potential mediators of the observed associations. Given the cross-sectional design, temporal relationships among the exposures, potential mediators, and outcome cannot be established. Therefore, the observed mediation patterns should not be interpreted as evidence of causal pathways or specific biological mechanisms. These findings should be considered hypothesis-generating and require further validation.

### 4.1 Potential biological explanations for the associations between LMIIs and DKD

Hyperglycemia is an important driver of both macrovascular and microvascular complications, mainly through its effects on endothelial function. Platelet dysfunction has also been implicated in the development of DKD in patients with T2DM. Under normal conditions, platelets remain quiescent and become activated mainly in response to vascular injury. However, hyperglycemia, insulin resistance, and elevated free fatty acids can activate platelet TLRs and the downstream MyD88/NF-κB pathway, thereby promoting platelet adhesion, activation, and aggregation (32). Activated platelets may further increase thrombosis, vascular permeability, and endothelial injury and facilitate leukocyte recruitment, thus sustaining local inflammation (33). Platelet recruitment can also promote the formation of neutrophil extracellular traps (NETs), which have been reported to activate the NOD-like receptor family pyrin domain containing 3 (NLRP3) inflammasome and aggravate glomerular endothelial cell injury in DKD (34, 35). In addition, activated platelets can bind to PSGL-1 on circulating monocytes, leading to the formation of monocyte - platelet aggregates (MPAs) (36). Hyperglycemia further facilitates monocyte adhesion to the endothelium by increasing the expression of adhesion molecules such as ICAM-1 and VCAM-1 (37). Some of these monocytes may subsequently migrate into the renal interstitium and differentiate into macrophages, which release inflammatory cytokines, including TNF-α and IL-1β, and contribute to tubulointerstitial fibrosis and glomerulosclerosis (38).

HDL has several anti-inflammatory and vascular-protective properties. It can suppress the expression of endothelial adhesion molecules, including E-selectin, ICAM-1, and VCAM-1, and inhibit NF-κB activation, thereby reducing leukocyte adhesion and migration (39–41). PON1, an enzyme associated with HDL, helps limit oxidative stress and LDL oxidation, while HDL itself promotes cholesterol efflux from macrophages and may suppress inflammasome activation (42–44). However, these protective functions of HDL are impaired in T2DM. PON1 activity is reduced in patients with T2DM (45), and glycation of ApoA-I can alter HDL function. Glycated HDL has been shown *in vitro* to activate NF-κB and increase cytokine production in macrophages, suggesting a shift toward a pro-inflammatory phenotype (46) Similarly, HDL from patients with CKD has a reduced capacity to inhibit VCAM-1 expression in endothelial cells (47).

PHR, NHR, MHR, and LHR are composite indicators of inflammation and lipid metabolism. Cellular indices such as the neutrophil-to-lymphocyte ratio (NLR) may reflect lipoinflammatory activation, although they remain nonspecific and context-dependent (48). The associations between LMIIs and prevalent DKD did not follow a simple linear pattern, with the strength of the association tending to plateau beyond a certain level. This pattern may be related to compensatory mechanisms within the body. When inflammation or lipid metabolic disturbances are mild to moderate, pathological processes, such as platelet activation, neutrophil infiltration, and monocyte chemotaxis, may progressively intensify and be involved in glomerular endothelial injury and tubulointerstitial fibrosis. However, once these indices exceed a certain level, negative feedback mechanisms may be activated; alternatively, in advanced disease stages, these pathways may no longer exhibit dose–response relationships. Statins and fibrates not only reduce lipid levels but also exert anti-inflammatory, antioxidant, and endothelial function - enhancing effects. Subgroup analyses suggested that lipid-lowering therapy may modify the association between LMIIs and prevalent DKD.

### 4.2 Clinical implications

In clinical practice, glycemic control and lipid management remain fundamental components of diabetes care. Our findings suggest that LMIIs, which can be readily calculated from routinely available blood cell counts and lipid parameters, may provide additional information on the lipid–inflammatory status of patients with T2DM and their likelihood of prevalent DKD. LMIIs may warrant further investigation as potential markers for DKD risk stratification, particularly in combination with established clinical and laboratory risk factors.

### 4.3 Limitations and outlook

However, several limitations should be acknowledged. First, the cross-sectional design limits causal inference and leaves open the possibility of reverse causation. Although higher PHR, NHR, MHR, and LHR levels were associated with greater odds of prevalent DKD, DKD itself may influence lipid metabolism and inflammatory status. Therefore, these associations, including the statistically inferred mediation effects, should not be interpreted as causal relationships. Second, despite excluding patients with overt acute diseases, unmeasured or subclinical inflammatory conditions may have contributed to residual confounding. Third, waist circumference was unavailable, precluding assessment of central adiposity and limiting evaluation of the independent effects of overall and central obesity. Fourth, the relatively small sample size and single-center design may limit generalizability, particularly given the limited representation of non-proteinuric renal phenotypes. Future multicenter prospective studies are warranted to validate these findings and clarify the temporal and causal relationships between LMIIs and DKD.

## 5 Conclusions

In summary, our findings revealed novel associations between LMIIs and DKD, suggesting that LMIIs may warrant further investigation as potential markers for DKD risk stratification.

## Data Availability

The original contributions presented in the study are included in the article/**Supplementary Material**. Further inquiries can be directed to the corresponding authors.
