# Supplementary material for: Lipid metabolism-related inflammatory indices and diabetic kidney disease in patients with type 2 diabetes mellitus: a cross-sectional study
Source: Front Endocrinol (Lausanne). 2026 Oct 13;17:1934556. doi: 10.3389/fendo.2026.1934556 (PMC13709990; doi:10.3389/fendo.2026.1934556)
Supplement: Supplementary file 1 [file SupplementaryFile1.docx]

Table S1. Missing number (%) for variables

| **Variables** | **Missing number (%)** |
| --- | --- |
| Age (years) | 0(0) |
| Diabetes duration (years) | 79(2.28) |
| FPG (mmol/L) | 0(0) |
| HbA1c (%) | 206(5.94) |
| CRP (mg/dL) | 0(0) |
| Hb (g/L) | 0(0) |
| ALB (g/L) | 0(0) |
| ALT (U/L) | 0(0) |
| AST (U/L) | 0(0) |
| LDL-C (mmol/L) | 0(0) |
| TG (mmol/L) | 0(0) |
| Sex, (n,%) | 0(0) |
| Ethnicity (n, %) | 0(0) |
| Drinking (n, %) | 0(0) |
| Smoking (n, %) | 0(0) |
| Hypertension (n, %) | 0(0) |
| CVD (n, %) | 0(0) |
| Obesity (n, %) | 0(0) |
| antidiabetic medications (n, %) | 0(0) |
| lipid-lowering agents (n, %) | 0(0) |
| Aspirin (n, %) | 0(0) |
| RAAS inhibitors (n, %) | 0(0) |

**Abbreviations**: FPG: Fasting Plasma Glucose; HbA1c: Glycosylated hemoglobin.; CRP: C-reactive protein; Hb: Hemoglobin; ALB: Albumin; ALT: Alanine Aminotransferase; AST: Aspartate Aminotransferase; LDL-C: Low-Density Lipoprotein Cholesterol; TG: Triglycerides; CVD: Cardiovascular Disease; RAAS inhibitors: Renin-Angiotensin-Aldosterone System inhibitors.

Table S2 Baseline characteristics of included and excluded patients

| **Characteristic** | **Overall** | **Included** | **Excluded** | ***P* value** |
| --- | --- | --- | --- | --- |
| N | 4149 | 3468 | 681 |  |
| Age (years) | 60.00 [52.00, 67.00] | 60.00 [52.00, 67.00] | 59.00 [50.00, 68.00] | 0.126 |
| Duration of diabetes (years) | 11.00 [6.00, 18.00] | 11.00 [6.00, 18.00] | 11.00 [5.00, 17.00] | 0.069 |
| FPG (mmol/L) | 10.60 [7.94, 14.40] | 10.55 [7.94, 14.40] | 10.89 [7.94, 14.54] | 0.395 |
| HbA1c (%) | 8.50 [7.30, 9.90] | 8.40 [7.30, 9.90] | 8.60 [7.30, 10.20] | 0.049 |
| CRP (mg/dL) | 1.00 [0.25, 2.36] | 1.00 [0.25, 2.32] | 1.01 [0.25, 2.71] | 0.001 |
| Hb (g/L) | 140.26 (17.04) | 140.06 (16.84) | 141.30 (18.01) | 0.098 |
| Scr (umol/L) | 61.00 [52.00, 73.10] | 61.00 [52.00, 74.00] | 61.10 [51.70, 72.00] | 0.117 |
| ALB (g/L) | 43.60 [41.30, 45.90] | 43.50 [41.30, 45.90] | 43.70 [41.40, 46.10] | 0.065 |
| ALT (U/L) | 19.80 [14.40, 29.00] | 19.50 [14.30, 28.50] | 21.60 [14.70, 32.30] | <0.001 |
| AST (U/L) | 19.50 [16.00, 25.00] | 19.45 [16.00, 25.00] | 19.80 [16.20, 25.90] | 0.147 |
| LDL-C (mmol/L) | 2.96 [2.37, 3.58] | 2.95 [2.37, 3.57] | 3.00 [2.39, 3.64] | 0.26 |
| TG (mmol/L) | 1.52 [1.10, 2.24] | 1.52 [1.10, 2.23] | 1.51 [1.11, 2.30] | <0.001 |
| TC (mmol/L) | 4.65 [3.86, 5.46] | 4.63 [3.85, 5.46] | 4.71 [3.92, 5.53] | 0.047 |
| Sex (n, %) |  |  |  | 0.106 |
| Male | 2201 (53.0) | 1820 (52.5) | 381 (55.9) |  |
| Female | 1948 (47.0) | 1648 (47.5) | 300 (44.1) |  |
| Ethnicity (n, %) |  |  |  | 0.627 |
| Han ethnicity | 3885 (93.6) | 3244 (93.5) | 641 (94.1) |  |
| Others | 264 (6.4) | 224 (6.5) | 40 (5.9) |  |
| Drinking (n, %) |  |  |  | 0.445 |
| No | 2555 (61.6) | 2145 (61.9) | 410 (60.2) |  |
| Yes | 1594 (38.4) | 1323 (38.1) | 271 (39.8) |  |
| Smoking (n, %) |  |  |  | 0.279 |
| No | 2529 (61.0) | 2127 (61.3) | 402 (59.0) |  |
| Yes | 1620 (39.0) | 1341 (38.7) | 279 (41.0) |  |
| Hypertension (n, %) |  |  |  | 0.543 |
| No | 1611 (38.8) | 1339 (38.6) | 272 (39.9) |  |
| Yes | 2538 (61.2) | 2129 (61.4) | 409 (60.1) |  |
| CVD (n, %) |  |  |  | 0.651 |
| No | 3020 (72.8) | 2519 (72.6) | 501 (73.6) |  |
| Yes | 1129 (27.2) | 949 (27.4) | 180 (26.4) |  |
| Obesity (n, %) |  |  |  | 0.193 |
| No | 3747 (90.3) | 3147 (90.7) | 600 (88.1) |  |
| Yes | 402 (9.7) | 321 (9.3) | 81 (11.9) |  |
| Antidiabetic medications (n, %) |  |  |  | 0.863 |
| No | 302 (7.3) | 254 (7.3) | 48 (7.0) |  |
| Yes | 3847 (92.7) | 3214 (92.7) | 633 (93.0) |  |
| Lipid-lowering agents (n, %) |  |  |  | 0.352 |
| No | 1311 (31.6) | 1085 (31.3) | 226 (33.2) |  |
| Yes | 2838 (68.4) | 2383 (68.7) | 455 (66.8) |  |
| Aspirin (n, %) |  |  |  | 0.266 |
| No | 2698 (65.0) | 2242 (64.6) | 456 (67.0) |  |
| Yes | 1451 (35.0) | 1226 (35.4) | 225 (33.0) |  |
| RAAS inhibitors (n, %) |  |  |  | 0.433 |
| No | 2764 (66.6) | 2301 (66.3) | 463 (68.0) |  |
| Yes | 1385 (33.4) | 1167 (33.7) | 218 (32.0) |  |

**Abbreviations:** FPG: Fasting Plasma Glucose; HbA1c: Glycosylated hemoglobin.; CRP: C-reactive protein; PHR: Platelet-to-high-density lipoprotein cholesterol ratio; NHR: neutrophil-to-high-density lipoprotein cholesterol ratio; MHR: monocyte-to-high-density lipoprotein cholesterol ratio; LHR: lymphocyte-to-high-density lipoprotein cholesterol ratio; Hb: Hemoglobin; Scr: Serum creatinine; eGFR: estimated glomerular filtration rate; UACR: urine albumin-to-creatinine ratio; ALB: Albumin; ALT: Alanine Aminotransferase; AST: Aspartate Aminotransferase; LDL-C: Low-Density Lipoprotein Cholesterol; TG: Triglycerides; CVD: Cardiovascular Disease; RAAS inhibitors: Renin-Angiotensin-Aldosterone System inhibitors.

Table S3 Associations of lipid metabolism-related inflammatory indices (LMIIs) with diabetic kidney disease (DKD)

| **Categories** | **Model 1**  **OR(95% CI)** | ***P-*value** | **Model 2**  **OR(95% CI)** | ***P-*value** | **Model 3**  **OR(95% CI)** | ***P-*value** |
| --- | --- | --- | --- | --- | --- | --- |
| Platelet count | 1.14 (1.01-1.29) | 0.03 | 1.33 (1.17–1.51) | <0.001 | 1.21 (1.05–1.40) | 0.007 |
| Platelet count (category) |  |  |  |  |  |  |
| Q1 (≤1.9) | Ref. |  | Ref. |  | Ref. |  |
| Q2 (1.9,2.23] | 1.00 (0.80-1.23) | 0.970 | 1.12 (0.90–1.39) | 0.314 | 1.06 (0.84–1.34) | 0.622 |
| Q3 (2.23,2.64] | 1.20 (0.97-1.48) | 0.091 | 1.41 (1.13–1.75) | 0.002 | 1.40 (1.10–1.77) | 0.005 |
| Q4 (＞2.64) | 1.26 (1.02-1.55) | 0.032 | 1.62 (1.30–2.01) | <0.001 | 1.41 (1.11–1.80) | 0.005 |
| *P* for trend |  | 0.010 |  | <0.001 |  | <0.001 |
| Neutrophil count | 1.30 (1.23-1.37) | <0.001 | 1.30 (1.23–1.37) | <0.001 | 1.27 (1.20–1.35) | <0.001 |
| (category) |  |  |  |  |  |  |
| Q1 (≤3.34) | Ref. |  | Ref. |  | Ref. |  |
| Q2 (3.34,4.11] | 1.49 (1.19-1.88) | <0.001 | 1.42 (1.12–1.79) | 0.004 | 1.33 (1.04–1.71) | 0.025 |
| Q3 (4.11,5.05] | 2.19 (1.75-2.74) | <0.001 | 2.09 (1.67–2.62) | <0.001 | 1.94 (1.52–2.48) | <0.001 |
| Q4 (＞5.05) | 2.69 (2.16-3.36) | <0.001 | 2.60 (2.08–3.26) | <0.001 | 2.25 (1.76–2.87) | <0.001 |
| *P* for trend |  | <0.001 |  | <0.001 |  | <0.001 |
| Monocyte count | 1.30 (1.22-1.38) | <0.001 | 1.27 (1.19–1.35) | <0.001 | 1.24 (1.15–1.33) | <0.001 |
| (category) |  |  |  |  |  |  |
| Q1 (≤2.8) | Ref. |  | Ref. |  | Ref. |  |
| Q2 (2.8,3.5] | 1.23 (0.99-1.53) | 0.057 | 1.20 (0.96–1.49) | 0.102 | 1.09 (0.86–1.37) | 0.497 |
| Q3 (3.5,4.3] | 1.75 (1.41-2.17) | <0.001 | 1.65 (1.32–2.06) | <0.001 | 1.45 (1.14-1.84) | 0.003 |
| Q4 (＞4.3) | 2.19 (1.76-2.72) | <0.001 | 2.01 (1.61-2.51) | <0.001 | 1.80 (1.41–2.30) | <0.001 |
| *P* for trend |  | <0.001 |  | <0.001 |  | <0.001 |
| Lymphocyte count | 1.00 (0.88-1.13) | 0.980 | 1.11 (0.98–1.26) | 0.109 | 1.20 (1.03–1.39) | 0.016 |
| (category) |  |  |  |  |  |  |
| Q1 (≤1.35) | Ref. |  | Ref. |  | Ref. |  |
| Q2 (1.35,1.69] | 0.90 (0.73-1.11) | 0.3420 | 0.98 (0.79–1.21) | 0.850 | 1.12 (0.89–1.41) | 0.339 |
| Q3 (1.69,2.08] | 0.91 (0.74-1.12) | 0.3520 | 1.03 (0.83–1.27) | 0.818 | 1.17 (0.92–1.49) | 0.189 |
| Q4 (＞2.08) | 0.96 (0.78-1.18) | 0.7030 | 1.14 (0.92–1.42) | 0.226 | 1.35 (1.06–1.72) | 0.016 |
| *P* for trend |  | 0.7154 |  | 0.205 |  | 0.016 |
| HDL-C | 0.58 (0.44-0.77) | <0.001 | 0.59 (0.44, 0.78) | <0.001 | 0.67 (0.47, 0.94) | 0.022 |
| (category) |  |  |  |  |  |  |
| Q1 (≤0.95) | Ref. |  | Ref. |  | Ref. |  |
| Q2 (0.95,1.1] | 0.82 (0.67-1.00) | 0.054 | 0.81 (0.66-0.99) | 0.045 | 0.93 (0.74-1.17) | 0.533 |
| Q3 (1.1,1.29] | 0.76 (0.62-0.93) | 0.009 | 0.75 (0.61-0.93) | 0.009 | 0.83 (0.65-1.05) | 0.118 |
| Q4 (＞1.29) | 0.66 (0.53-0.81) | <0.001 | 0.66 (0.53-0.82) | <0.001 | 0.72 (0.56-0.94) | 0.015 |
| *P* for trend |  | <0.001 |  | <0.001 |  | 0.011 |
| PHR | 1.22(1.11, 1.34) | <0.001 | 1.33 (1.20, 1.46) | <0.001 | 1.20 (1.07, 1.34) | 0.001 |
| PHR (category) |  |  |  |  |  |  |
| Q1 (≤1.59) | Ref. |  | Ref. |  | Ref. |  |
| Q2 (1.59, 2.01] | 1.35(1.08, 1.67) | 0.007 | 1.39 (1.11, 1.73) | 0.004 | 1.38 (1.09-1.75) | 0.008 |
| Q3 (2.01, 2.51] | 1.38 (1.12, 1.72) | 0.003 | 1.50(1.21, 1.87) | <0.001 | 1.50 (1.18-1.91) | <0.001 |
| Q4 (>2.51) | 1.66 (1.35, 2.06) | <0.001 | 1.93 (1.55, 2.41) | <0.001 | 1.66 (1.29-2.12) | <0.001 |
| *P* for trend |  | <0.001 |  | <0.001 |  | <0.001 |
| NHR | 1.22 (1.17, 1.27) | <0.001 | 1.22 (1.17, 1.27) | <0.001 | 1.20 (1.14, 1.26) | <0.001 |
| NHR (category) |  |  |  |  |  |  |
| Q1 (≤2.79) | Ref. |  | Ref. |  | Ref. |  |
| Q2 (2.79, 3.72] | 1.30 (1.04, 1.64) | 0.025 | 1.25 (0.99, 1.58) | 0.059 | 1.21 (0.94, 1.55) | 0.144 |
| Q3 (3.72, 4.93] | 1.96 (1.58, 2.45) | <0.001 | 1.92 (1.54, 2.41) | <0.001 | 1.82 (1.42, 2.33) | <0.001 |
| Q4 (>4.93) | 2.70 (2.18, 3.36) | <0.001 | 2.63 (2.11, 3.28) | <0.001 | 2.35 (1.82, 3.03) | <0.001 |
| *P* for trend |  | <0.001 |  | <0.001 |  | <0.001 |
| MHR | 1.21 (1.16, 1.27) | <0.001 | 1.21 (1.15, 1.27) | <0.001 | 1.18 (1.11, 1.26) | <0.001 |
| MHR (category) |  |  |  |  |  |  |
| Q1 (≤2.38) | Ref. |  | Ref. |  | Ref. |  |
| Q2 (2.38, 3.15] | 1.29 (1.03, 1.62) | 0.026 | 1.28 (1.02, 1.61) | 0.036 | 1.18 (0.92, 1.51) | 0.200 |
| Q3 (3.15, 4.10] | 1.86 (1.50, 2.32) | <0.001 | 1.79 (1.43, 2.24) | <0.001 | 1.64 (1.28, 2.11) | <0.001 |
| Q4 (>4.10) | 2.28 (1.84, 2.83) | <0.001 | 2.25 (1.79, 2.83) | <0.001 | 2.00 (1.54, 2.60) | <0.001 |
| *P* for trend |  | <0.001 |  | <0.001 |  | <0.001 |
| LHR | 1.09 (0.99, 1.20) | 0.089 | 1.18 (1.07, 1.31) | 0.002 | 1.21 (1.07, 1.37) | 0.003 |
| LHR (category) |  |  |  |  |  |  |
| Q1 (≤1.13) | Ref. |  | Ref. |  | Ref. |  |
| Q2 (1.13, 1.52] | 1.08 (0.87, 1.34) | 0.481 | 1.12 (0.90, 1.38) | 0.319 | 1.11 (0.88, 1.41) | 0.387 |
| Q3 (1.52, 2.00] | 1.20 (0.98, 1.48) | 0.085 | 1.31 (1.06, 1.63) | 0.013 | 1.43 (1.13, 1.82) | 0.003 |
| Q4 (>2.00) | 1.21 (0.98, 1.50) | 0.071 | 1.40 (1.13, 1.74) | 0.002 | 1.49 (1.16, 1.92) | 0.002 |
| *P* for trend |  | 0.042 |  | <0.001 |  | <0.001 |

Model 1 was unadjusted.

Model 2 was adjusted for age, sex, ethnicity, and diabetes duration.

Model 3 was adjusted for age, sex, ethnicity, and diabetes duration, FPG, HbA1c, CRP, Hb, ALB, ALT, AST, LDL-C, TG, hypertension, CVD, obesity, antidiabetic medications, lipid-lowering agents, aspirin, RAAS inhibitors, smoking and drinking.

**Abbreviations:** FPG: Fasting Plasma Glucose; HbA1c: Glycosylated hemoglobin.; CRP: C-reactive protein; Hb: Hemoglobin; ALB: Albumin; ALT: Alanine Aminotransferase; AST: Aspartate Aminotransferase; LDL-C: Low-Density Lipoprotein Cholesterol; TG: Triglycerides; CVD: Cardiovascular Disease; RAAS inhibitors: Renin-Angiotensin-Aldosterone System inhibitors.

Table S4. Associations between lipid metabolism-related inflammatory indices (LMIIs) and diabetic kidney disease (DKD) using inverse probability of treatment weighting (IPTW) method

| Character | OR(95%CI) | *P* |
| --- | --- | --- |
| PHR category |  |  |
| Lower PHR | ref |  |
| Higher PHR | 1.35(1.13, 1.60) | <0.001 |
| NHR category |  |  |
| Lower NHR | ref |  |
| Higher NHR | 1.86(1.56, 2.22) | <0.001 |
| MHR category |  |  |
| Lower MHR | ref |  |
| Higher MHR | 1.61(1.34, 1.93) | <0.001 |
| LHR category |  |  |
| Lower LHR | ref |  |
| Higher LHR | 1.35(1.14, 1.61) | <0.001 |

**Abbreviations:** PHR: Platelet-to-high-density lipoprotein cholesterol ratio; NHR: neutrophil-to-high-density lipoprotein cholesterol ratio; MHR: monocyte-to-high-density lipoprotein cholesterol ratio; LHR: lymphocyte-to-high-density lipoprotein cholesterol ratio.

Table S5. Distribution of inverse probability weights before and after truncation

| Characteristic | Original weights | Truncated weights |
| --- | --- | --- |
| PHR |  |  |
| n | 3468 | 3468 |
| Mean | 1.029 | 0.989 |
| Median | 0.921 | 0.921 |
| Range (min, max) | 0.501，72.884 | 0.552，2.427 |
| 1st percentile | 0.552 | — |
| 99th percentile | 2.427 | — |
| Truncation proportion (%) | — | 2.02% |
| Effective sample size | 1,192.5 | 3,151.2 |
| NHR |  |  |
| n | 3468 | 3468 |
| Mean | 1.288 | 0.983 |
| Median | 0.873 | 0.873 |
| Range (min, max) | 0.500，519.386 | 0.518，3.173 |
| 1st percentile | 0.518 | — |
| 99th percentile | 3.173 | — |
| Truncation proportion (%) | — | 2.02% |
| Effective sample size | 54.2 | 2,950.8 |
| MHR |  |  |
| n | 3468 | 3468 |
| Mean | 1.386 | 0.983 |
| Median | 0.823 | 0.823 |
| Range (min, max) | 0.501，1093.456 | 0.517，3.604 |
| 1st percentile | 0.517 | — |
| 99th percentile | 3.604 | — |
| Truncation proportion (%) | — | 2.02% |
| Effective sample size | 18.5 | 2,729.5 |
| LHR |  |  |
| n | 3468 | 3468 |
| Mean | 1.549 | 0.979 |
| Median | 0.885 | 0.885 |
| Range (min, max) | 0.500，1370.264 | 0.520，2.696 |
| 1st percentile | 0.520 | — |
| 99th percentile | 2.696 | — |
| Truncation proportion (%) | — | 2.02% |
| Effective sample size | 13.6 | 3,048.3 |

**Abbreviations:** PHR: Platelet-to-high-density lipoprotein cholesterol ratio; NHR: neutrophil-to-high-density lipoprotein cholesterol ratio; MHR: monocyte-to-high-density lipoprotein cholesterol ratio; LHR: lymphocyte-to-high-density lipoprotein cholesterol ratio.

Table S6. Standardized mean differences (SMD) before and after nverse probability of treatment weighting (IPTW)

| **Variable** | **PHR** |  | **NHR** |  | **MHR** |  | **LHR** |  |
| --- | --- | --- | --- | --- | --- | --- | --- | --- |
|  | **Unw.SMD** | **Wtd.SMD** | **Unw.SMD** | **Wtd.SMD** | **Unw.SMD** | **Wtd.SMD** | **Unw.SMD** | **Wtd.SMD** |
| Sex | -0.052 | -0.001 | -0.125 | -0.009 | -0.265 | -0.016 | -0.082 | -0.005 |
| Age | -0.339 | -0.032 | -0.042 | -0.001 | -0.032 | -0.015 | -0.389 | -0.044 |
| Ethnicity | -0.003 | -0.001 | 0.006 | 0.000 | 0.020 | 0.001 | -0.006 | 0.001 |
| Smoking | -0.005 | -0.002 | 0.037 | -0.002 | 0.025 | 0.002 | 0.011 | -0.002 |
| Drinking | 0.009 | 0.003 | 0.016 | 0.003 | 0.013 | 0.002 | 0.016 | -0.001 |
| Duration of diabetes | -0.126 | -0.013 | 0.002 | 0.001 | 0.002 | 0.016 | -0.103 | -0.008 |
| FBG | 0.052 | 0.002 | 0.149 | -0.004 | 0.084 | -0.007 | -0.051 | -0.005 |
| HbA1c | 0.121 | 0.008 | 0.049 | -0.002 | 0.052 | -0.004 | 0.073 | 0.008 |
| HB | 0.072 | 0.001 | 0.133 | 0.004 | 0.259 | 0.017 | 0.323 | 0.029 |
| CRP | 0.138 | 0.002 | 0.245 | 0.054 | 0.205 | 0.044 | 0.013 | 0.000 |
| ALB | 0.032 | -0.004 | -0.082 | -0.014 | -0.098 | -0.018 | 0.189 | 0.022 |
| ALT | 0.111 | 0.023 | 0.003 | -0.002 | 0.134 | 0.012 | 0.199 | 0.031 |
| AST | -0.030 | 0.003 | -0.096 | 0.002 | 0.064 | 0.010 | 0.073 | 0.015 |
| LDL | -0.156 | -0.012 | -0.317 | -0.027 | -0.419 | -0.024 | -0.189 | -0.018 |
| TG | 0.316 | 0.036 | 0.347 | 0.020 | 0.323 | 0.022 | 0.414 | 0.065 |
| Hypertension | 0.020 | -0.003 | 0.101 | 0.011 | 0.076 | 0.005 | -0.006 | -0.007 |
| CVD | -0.034 | -0.004 | 0.084 | 0.008 | 0.088 | 0.012 | -0.005 | 0.000 |
| Obesity | 0.055 | 0.006 | 0.043 | 0.004 | 0.044 | 0.006 | 0.058 | 0.007 |
| Antidiabetic medications | 0.005 | -0.001 | 0.012 | 0.000 | 0.003 | 0.000 | 0.005 | 0.002 |
| Lipid-lowering agents | 0.001 | -0.002 | 0.007 | 0.005 | -0.025 | 0.001 | -0.014 | -0.003 |
| Aspirin | 0.003 | 0.001 | 0.015 | 0.002 | 0.001 | 0.006 | -0.002 | -0.001 |
| RAAS inhibitors | -0.032 | -0.002 | 0.006 | -0.002 | -0.008 | -0.005 | -0.005 | 0.001 |
| Mean SMD | **0.078** | **0.007** | **0.087** | **0.008** | **0.102** | **0.011** | **0.101** | **0.013** |

**Abbreviations:** PHR: Platelet-to-high-density lipoprotein cholesterol ratio; NHR: neutrophil-to-high-density lipoprotein cholesterol ratio; MHR: monocyte-to-high-density lipoprotein cholesterol ratio; LHR: lymphocyte-to-high-density lipoprotein cholesterol ratio; SMD: standardized mean difference; Mean SMD: mean absolute standardized mean difference.

Table S7. Sensitivity analysis of the associations between lipid metabolism-related inflammatory indices (LMIIs) and diabetic kidney disease (DKD) after adjustment for continuous BMI

| **Categories** | **Model**  **OR(95% CI)** | ***P-*value** |
| --- | --- | --- |
| PHR | 1.16 (1.04, 1.30) | 0.008 |
| PHR (category) |  |  |
| Q1 (≤1.59) | Ref. |  |
| Q2 (1.59, 2.01] | 1.35(1.06, 1.72) | 0.015 |
| Q3 (2.01, 2.51] | 1.44 (1.13, 1.83) | 0.003 |
| Q4 (>2.51) | 1.56 (1.22, 2.00) | <0.001 |
| *P* for trend |  | <0.001 |
| NHR | 1.19 (1.13, 1.25) | <0.001 |
| NHR (category) |  |  |
| Q1 (≤2.79) | Ref. |  |
| Q2 (2.79, 3.72] | 1.18 (0.92-1.52) | 0.192 |
| Q3 (3.72, 4.93] | 1.74 (1.36-2.23) | <0.001 |
| Q4 (>4.93) | 2.22 (1.72-2.86) | <0.001 |
| P for trend |  | <0.001 |
| MHR | 1.17 (1.10, 1.24) | <0.001 |
| MHR (category) |  |  |
| Q1 (≤2.38) | Ref. |  |
| Q2 (2.38, 3.15] | 1.13 (0.88-1.46) | 0.330 |
| Q3 (3.15, 4.10] | 1.55 (1.21-2.00) | <0.001 |
| Q4 (>4.10) | 1.87 (1.43-2.43) | <0.001 |
| P for trend |  | <0.001 |
| LHR | 1.18 (1.04, 1.34) | 0.011 |
| LHR (category) |  |  |
| Q1 (≤1.13) | Ref. |  |
| Q2 (1.13, 1.52] | 1.08 (0.85-1.37) | 0.524 |
| Q3 (1.52, 2.00] | 1.36 (1.07-1.73) | 0.012 |
| Q4 (>2.00) | 1.42 (1.11-1.83) | 0.006 |
| P for trend |  | 0.001 |

Model was adjusted for age, sex, ethnicity, and diabetes duration, FPG, HbA1c, CRP, Hb, ALB, ALT, AST, LDL-C, TG, hypertension, CVD, BMI(continuous), antidiabetic medications, lipid-lowering agents, aspirin, RAAS inhibitors, smoking and drinking.

**Abbreviations:** PHR: Platelet-to-high-density lipoprotein cholesterol ratio; NHR: neutrophil-to-high-density lipoprotein cholesterol ratio; MHR: monocyte-to-high-density lipoprotein cholesterol ratio; LHR: lymphocyte-to-high-density lipoprotein cholesterol ratio.

Table S8 Sensitivity analysis of the association between lipid metabolism-related inflammatory indices (LMIIs) and diabetic kidney disease (DKD) based on repeated measurements

| **Categories** | **Model 1**  **OR(95% CI)** | ***P-*value** | **Model 2**  **OR(95% CI)** | ***P-*value** | **Model 3**  **OR(95% CI)** | ***P-*value** |
| --- | --- | --- | --- | --- | --- | --- |
| PHR | 1.31 (1.16, 1.49) | <0.001 | 1.43 (1.26, 1.64) | <0.001 | 1.31 (1.12, 1.53) | <0.001 |
| PHR (category) |  |  |  |  |  |  |
| Q1 (≤1.60) | Ref. |  | Ref. |  | Ref. |  |
| Q2 (1.60, 2.01] | 1.62 (1.22, 2.17) | 0.001 | 1.69 (1.26, 2.27) | <0.001 | 1.55 (1.13, 2.15) | 0.007 |
| Q3 (2.01, 2.49] | 1.52 (1.13, 2.03) | 0.005 | 1.66 (1.23, 2.24) | <0.001 | 1.55 (1.12, 2.16) | 0.009 |
| Q4 (>2.49) | 1.99 (1.50, 2.66) | <0.001 | 2.33 (1.74, 3.13) | <0.001 | 1.94 (1.39, 2.71) | <0.001 |
| *P* for trend |  | <0.001 |  | <0.001 |  | 0.001 |
| NHR | 1.22 (1.16, 1.29) | <0.001 | 1.23 (1.17, 1.30) | <0.001 | 1.23 (1.15, 1.31) | <0.001 |
| NHR (category) |  |  |  |  |  |  |
| Q1 (≤2.79) | Ref. |  | Ref. |  | Ref. |  |
| Q2 (2.79, 3.76] | 1.40 (1.03, 1.91) | 0.030 | 1.34 (0.98, 1.82) | 0.067 | 1.30 (0.93, 1.83) | 0.125 |
| Q3 (3.76, 4.99] | 2.04 (1.52, 2.75) | <0.001 | 2.02 (1.50, 2.73) | <0.001 | 2.07 (1.48, 2.89) | <0.001 |
| Q4 (>4.99) | 2.91 (2.19, 3.91) | <0.001 | 2.92 (2.18, 3.94) | <0.001 | 2.58 (1.84, 3.63) | <0.001 |
| P for trend |  | <0.001 |  | <0.001 |  | <0.001 |
| MHR | 1.21 (1.14, 1.29) | <0.001 | 1.21 (1.14, 1.30) | <0.001 | 1.20 (1.11, 1.30) | <0.001 |
| MHR (category) |  |  |  |  |  |  |
| Q1 (≤2.37) | Ref. |  | Ref. |  | Ref. |  |
| Q2 (2.37, 3.15] | 1.17 (0.87, 1.57) | 0.308 | 1.11 (0.82, 1.50) | 0.491 | 1.07 (0.77, 1.49) | 0.694 |
| Q3 (3.15, 4.07] | 1.68 (1.26, 2.24) | <0.001 | 1.62 (1.21, 2.18) | 0.001 | 1.59 (1.14, 2.22) | 0.006 |
| Q4 (>4.07) | 2.04 (1.54, 2.71) | <0.001 | 1.98 (1.47, 2.67) | <0.001 | 1.75 (1.24, 2.48) | 0.002 |
| P for trend |  | <0.001 |  | <0.001 |  | 0.001 |
| LHR | 1.14 (0.99, 1.31) | 0.064 | 1.24 (1.08, 1.44) | 0.003 | 1.28 (1.07, 1.51) | 0.005 |
| LHR (category) |  |  |  |  |  |  |
| Q1 (≤1.13) | Ref. |  | Ref. |  | Ref. |  |
| Q2 (1.13, 1.51] | 1.25 (0.94, 1.66) | 0.129 | 1.31 (0.99, 1.75) | 0.063 | 1.28 (0.93, 1.76) | 0.133 |
| Q3 (1.51, 1.98] | 1.35 (1.02, 1.79) | 0.038 | 1.48 (1.11, 1.98) | 0.007 | 1.56 (1.13, 2.16) | 0.007 |
| Q4 (>1.98) | 1.44 (1.09, 1.91) | 0.011 | 1.66 (1.24, 2.22) | <0.001 | 1.73 (1.24, 2.42) | 0.001 |
| P for trend |  | 0.010 |  | <0.001 |  | 0.002 |

Model was adjusted for age, sex, ethnicity, and diabetes duration, FPG, HbA1c, CRP, Hb, ALB, ALT, AST, LDL-C, TG, hypertension, CVD, obesity, antidiabetic medications, lipid-lowering agents, aspirin, RAAS inhibitors, smoking and drinking. Repeated measurements included urinary albumin-to-creatinine ratio (UACR) and/or estimated glomerular filtration rate (eGFR).

**Abbreviations:** PHR: Platelet-to-high-density lipoprotein cholesterol ratio; NHR: neutrophil-to-high-density lipoprotein cholesterol ratio; MHR: monocyte-to-high-density lipoprotein cholesterol ratio; LHR: lymphocyte-to-high-density lipoprotein cholesterol ratio.
