# Supplementary material for: Lipid metabolism-related inflammatory indices and diabetic kidney disease in patients with type 2 diabetes mellitus: a cross-sectional study
Source: Front Endocrinol (Lausanne). 2026 Oct 13;17:1934556. doi: 10.3389/fendo.2026.1934556 (PMC13709990; doi:10.3389/fendo.2026.1934556)
Supplement: Supplementary file 2 [file SupplementaryFile2.docx]

TableS9 Mediation analyses of potential mediators in the associations between lipid metabolism-related inflammatory indices (LMIIs) with diabetic kidney disease (DKD)

| **Mediators** | **Natural direct effect** | |  | | **Natural mediated effect** | | |  | **Total effect** | | | | **Proportion of mediating effect** | |  |
| --- | --- | --- | --- | --- | --- | --- | --- | --- | --- | --- | --- | --- | --- | --- | --- |
|  | **Effect estimate (95% CI)** | ***P* value** |  | | **Effect estimate (95% CI)** | ***P* value** | |  | **Effect estimate (95% CI)** | | ***P* value** | |  |  |  |
| **PHR** (10^11^/mmol) |  |  |  | |  |  | |  |  | |  | |  | |  |
| HbA1c(%) | 0.036 (0.022, 0.051) | <0.001 |  | | 0.002 (0.000, 0.003) | 0.018 | |  | | 0.038 (0.024, 0.052) | <0.001 | | 4.73% | |  |
| FPG(mmol/L) | 0.036 (0.022, 0.050) | <0.001 |  | | 0.002 (0.000, 0.004) | 0.059 | |  | | 0.038 (0.024, 0.052) | <0.001 | | 4.62% | |  |
| LDL-C(mmol/L) | 0.045 (0.031, 0.059) | <0.001 |  | | -0.007 (-0.010,-0.004) | <0.001 | |  | | 0.038 (0.024, 0.052) | <0.001 | | -17.65% | |  |
| TG (mmol/L) | 0.030 (0.015, 0.045) | <0.001 |  | | 0.008 (0.005, 0.012) | <0.001 | |  | | 0.038 (0.024, 0.052) | <0.001 | | 21.14% | |  |
| CRP(mg/dL) | 0.035 (0.020, 0.050) | <0.001 |  | | 0.003 (0.000, 0.006) | 0.080 | |  | | 0.038 (0.024, 0.052) | <0.001 | | 7.77% | |  |
| Hb (g/L) | 0.033 (0.018, 0.047) | <0.001 |  | | 0.006 (0.003, 0.009) | <0.001 | |  | | 0.038 (0.024, 0.052) | <0.001 | | 15.06% | |  |
| ALB(g/L) | 0.031 (0.017, 0.045) | <0.001 |  | | 0.007 (0.003, 0.011) | 0.001 | |  | | 0.038 (0.024, 0.052) | <0.001 | | 18.54% | |  |
| ALT (U/L) | 0.038 (0.024, 0.052) | <0.001 |  | | 0.000 (-0.001, 0.000) | 0.955 | |  | | 0.038 (0.024, 0.052) | <0.001 | | -0.04% | |  |
| AST (U/L) | 0.039 (0.025, 0.053) | <0.001 |  | | 0.000 (-0.002, 0.001) | 0.499 | |  | 0.038 (0.024, 0.052) | | <0.001 | | -1.19% | |  |
| **NHR** (10^9^/mmol) |  |  |  | |  |  | |  |  | |  | |  | |  |
| HbA1c(%) | 0.025 (0.020, 0.029) | <0.001 |  | | 0.001 (0.000, 0.001) | 0.029 | |  | 0.025 (0.021, 0.030) | | <0.001 | | 2.39% | |  |
| FPG(mmol/L) | 0.023 (0.019, 0.028) | <0.001 |  | | 0.002 (0.001, 0.003) | <0.001 | |  | 0.025 (0.021, 0.030) | | <0.001 | | 6.61% | |  |
| LDL-C(mmol/L) | 0.029 (0.024, 0.033) | <0.001 |  | | -0.004 (-0.005,-0.002) | <0.001 | |  | 0.025 (0.021, 0.030) | | <0.001 | | -13.88% | |  |
| TG (mmol/L) | 0.022 (0.017, 0.027) | <0.001 |  | | 0.003 (0.002, 0.005) | <0.001 | |  | 0.025 (0.021, 0.030) | | <0.001 | | 12.26% | |  |
| CRP(mg/dL) | 0.025 (0.020, 0.029) | <0.001 |  | | 0.000 (-0.002, 0.003) | 0.632 | |  | 0.025 (0.021, 0.030) | | <0.001 | | 1.98% | |  |
| Hb (g/L) | 0.025 (0.020, 0.029) | <0.001 |  | | 0.000 (-0.001, 0.001) | 0.793 | |  | 0.025 (0.020, 0.030) | | <0.001 | | 0.60% | |  |
| ALB(g/L) | 0.022 (0.017, 0.027) | <0.001 |  | 0.003 (0.002, 0.005) | | | <0.001 |  | 0.025 (0.021, 0.030) | | | <0.001 | | 12.44% | |
| ALT (U/L) | 0.025 (0.021, 0.030) | <0.001 |  | 0.000 (0.000, 0.000) | | | 0.945 |  | 0.025 (0.021, 0.030) | | | <0.001 | | 0.02% | |
| AST (U/L) | 0.025 (0.021, 0.030) | <0.001 |  | 0.000 (-0.001, 0.000) | | | 0.459 |  | 0.025 (0.021, 0.030) | | | <0.001 | | -0.58% | |
| **MHR** (10^8^/mmol) |  |  |  |  | | |  |  |  | | |  | |  | |
| HbA1c(%) | 0.025 (0.018, 0.032) | <0.001 |  | 0.001 (0.000, 0.001) | | | 0.043 |  | 0.026 (0.019, 0.032) | | | <0.001 | | 2.82% | |
| FPG(mmol/L) | 0.025 (0.018, 0.031) | <0.001 |  | 0.001 (0.000, 0.002) | | | 0.026 |  | 0.026 (0.019, 0.032) | | | <0.001 | | 4.25% | |
| LDL-C(mmol/L) | 0.031 (0.024, 0.037) | <0.001 |  | -0.005 (-0.007, -0.003) | | | <0.001 |  | 0.026 (0.019, 0.032) | | | <0.001 | | -19.66% | |
| TG (mmol/L) | 0.021 (0.014, 0.028) | <0.001 |  | 0.004 (0.002, 0.006) | | | <0.001 |  | 0.026 (0.019, 0.032) | | | <0.001 | | 16.66% | |
| CRP(mg/dL) | 0.024 (0.017, 0.031) | <0.001 |  | 0.001 (-0.001, 0.004) | | | 0.304 |  | 0.026 (0.019, 0.032) | | | <0.001 | | 5.26% | |
| Hb (g/L) | 0.026 (0.019, 0.033) | <0.001 |  | 0.000 (-0.002, 0.001) | | | 0.748 |  | 0.025 (0.019, 0.032) | | | <0.001 | | -0.97% | |
| ALB(g/L) | 0.021 (0.014, 0.028) | <0.001 |  | 0.005 (0.003, 0.007) | | | <0.001 |  | 0.026 (0.019, 0.033) | | | <0.001 | | 19.58% | |
| ALT (U/L) | 0.026 (0.019, 0.033) | <0.001 |  | 0.000 (-0.001, 0.001) | | | 0.459 |  | 0.026 (0.019, 0.032) | | | <0.001 | | -1.32% | |
| AST (U/L) | 0.026 (0.019, 0.032) | <0.001 |  | 0.000 (0.000, 0.000) | | | 0.784 |  | 0.026 (0.019, 0.032) | | | <0.001 | | 0.20% | |
| **LHR** (10^9^/mmol) |  |  |  |  | | |  |  |  | | |  | |  | |
| HbA1c(%) | 0.022 (0.004, 0.041) | 0.018 |  | 0.002 (0.000, 0.003) | | | 0.056 |  | 0.024 (0.006, 0.043) | | | 0.011 | | 6.82% | |
| FPG(mmol/L) | 0.026 (0.008, 0.044) | 0.006 |  | -0.002 (-0.004, 0.000) | | | 0.127 |  | 0.024 (0.006, 0.043) | | | 0.011 | | -6.59% | |
| LDL-C(mmol/L) | 0.032 (0.013, 0.050) | <0.001 |  | -0.007 (-0.011, -0.003) | | | <0.001 |  | 0.025 (0.006, 0.044) | | | 0.010 | | -27.88% | |
| TG (mmol/L) | 0.010 (-0.011,0.030) | 0.350 |  | 0.014 (0.008, 0.020) | | | <0.001 |  | 0.024 (0.005, 0.043) | | | 0.012 | | 59.42% | |
| CRP(mg/dL) | 0.023 (0.005, 0.042) | 0.014 |  | 0.001 (-0.001, 0.003) | | | 0.221 |  | 0.024 (0.006, 0.043) | | | 0.011 | | 4.50% | |
| Hb (g/L) | 0.035 (0.017, 0.053) | <0.001 |  | -0.010 (-0.015, -0.005) | | | <0.001 |  | 0.025 (0.006, 0.044) | | | 0.009 | | -40.23% | |
| ALB(g/L) | 0.028 (0.010, 0.046) | 0.003 |  | -0.002 (-0.008, 0.004) | | | 0.533 |  | 0.026 (0.007, 0.045) | | | 0.008 | | -7.83% | |
| ALT (U/L) | 0.025 (0.006, 0.043) | 0.010 |  | -0.001 (-0.002, 0.001) | | | 0.594 |  | 0.024 (0.005, 0.043) | | | 0.011 | | -2.16% | |
| AST (U/L) | 0.024 (0.006, 0.043) | 0.010 |  | 0.000 (0.000, 0.000) | | | 0.961 |  | 0.024 (0.006, 0.043) | | | 0.010 | | -0.05% | |

Adjusted for sex, age, ethnicity, smoking and drinking use, diabetes duration, obesity, history of hypertension, history of CVD and medication use (antidiabetic medications, lipid-lowering agents, aspirin, and RAAS inhibitors).

**Abbreviations:** LMII, lipid metabolism-related inflammatory indices; DKD, peripheral arterial disease; NHR, neutrophil to high-density lipoprotein cholesterol ratio; MHR, monocyte to high-density lipoprotein cholesterol ratio; PHR, platelet to high-density lipoprotein cholesterol ratio; LHR, lymphocyte to high-density lipoprotein cholesterol ratio;FPG: Fasting Plasma Glucose; HbA1c: Glycosylated hemoglobin.; CRP: C-reactive protein; Hb: Hemoglobin; ALB: Albumin; ALT: Alanine Aminotransferase; AST: Aspartate Aminotransferase; LDL-C: Low-Density Lipoprotein Cholesterol; TG: Triglycerides; CVD: Cardiovascular Disease; RAAS inhibitors: Renin-Angiotensin-Aldosterone System inhibitors.

Table S10 Proportions mediated in the associations between lipid metabolism-related inflammatory indices (LMIIs) with diabetic kidney disease (DKD)

| **Mediators** | **Proportion of mediating effect** | | |
| --- | --- | --- | --- |
|  | **Proportion mediated (95% CI)** | ***P* value** | **Proportion of mediating effect（%）** |
| **PHR** (10^11^/mmol) **^a^** |  |  |  |
| HbA1c(%) | 0.047 (0.000, 0.095) | 0.052 | 4.73% |
| FPG(mmol/L) | 0.046 (-0.009, 0.101) | 0.099 | 4.62% |
| LDL-C (mmol/L) | -0.176 (-0.310, -0.043) | 0.010 | -17.65% |
| TG (mmol/L) | 0.211 (0.054, 0.369) | 0.009 | 21.14% |
| CRP(mg/dL) | 0.078 (-0.025, 0.181) | 0.139 | 7.77% |
| Hb (g/L) | 0.151 (0.046, 0.255) | 0.005 | 15.06% |
| ALB(g/L) | 0.185 (0.047, 0.323) | 0.009 | 18.54% |
| ALT (U/L) | 0.000 (-0.015, 0.014) | 0.958 | -0.04% |
| AST (U/L) | -0.012 (-0.050, 0.026) | 0.542 | -1.19% |
| **NHR** (10^9^/mmol) **^a^** |  |  |  |
| HbA1c(%) | 0.024 (0.001, 0.046) | 0.037 | 2.39% |
| FPG(mmol/L) | 0.066 (0.027, 0.105) | <0.001 | 6.61% |
| LDL-C (mmol/L) | -0.139 (-0.204, -0.073) | <0.001 | -13.88% |
| TG (mmol/L) | 0.123 (0.059, 0.186) | <0.001 | 12.26% |
| CRP(mg/dL) | 0.020 (-0.063, 0.103) | 0.640 | 1.98% |
| Hb (g/L) | 0.006 (-0.040, 0.052) | 0.798 | 0.60% |
| ALB(g/L) | 0.124 (0.056, 0.193) | <0.001 | 12.44% |
| ALT (U/L) | 0.000 (-0.007, 0.007) | 0.946 | 0.02% |
| AST (U/L) | -0.006 (-0.022, 0.010) | 0.476 | -0.58% |
| **MHR** (10^8^/mmol) **^a^** |  |  |  |
| HbA1c(%) | 0.028 (-0.001, 0.058) | 0.061 | 2.82% |
| FPG(mmol/L) | 0.043 (0.003, 0.082) | 0.035 | 4.25% |
| LDL-C (mmol/L) | -0.197 (-0.305, -0.089) | <0.001 | -19.66% |
| TG (mmol/L) | 0.167 (0.072, 0.261) | <0.001 | 16.66% |
| CRP(mg/dL) | 0.053 (-0.055, 0.160) | 0.336 | 5.26% |
| Hb (g/L) | -0.010 (-0.073, 0.053) | 0.763 | -0.97% |
| ALB(g/L) | 0.196 (0.096, 0.295) | <0.001 | 19.58% |
| ALT (U/L) | -0.013 (-0.051, 0.024) | 0.490 | -1.32% |
| AST (U/L) | 0.002 (-0.013, 0.017) | 0.796 | 0.20% |
| **LHR** (10^9^/mmol) **^a^** |  |  |  |
| HbA1c(%) | 0.068 (-0.020, 0.156) | 0.128 | 6.82% |
| FPG(mmol/L) | -0.066 (-0.165, 0.033) | 0.191 | -6.59% |
| LDL-C (mmol/L) | -0.279 (-0.541, -0.016) | 0.037 | -27.88% |
| TG (mmol/L) | 0.594 (0.070, 1.118) | 0.026 | 59.42% |
| CRP(mg/dL) | 0.045 (-0.035, 0.125) | 0.270 | 4.50% |
| Hb (g/L) | -0.402 (-0.756, -0.048) | 0.026 | -40.23% |
| ALB(g/L) | -0.078 (-0.331, 0.175) | 0.544 | -7.83% |
| ALT (U/L) | -0.022 (-0.103, 0.059) | 0.602 | -2.16% |
| AST (U/L) | 0.000 (-0.020, 0.019) | 0.961 | -0.05% |

**Abbreviations:** LMIIs, lipid metabolism-related inflammatory indices; DKD, peripheral arterial disease; NHR, neutrophil to high-density lipoprotein cholesterol ratio; MHR, monocyte to high-density lipoprotein cholesterol ratio; PHR, platelet to high-density lipoprotein cholesterol ratio; LHR, lymphocyte to high-density lipoprotein cholesterol ratio; HbA1c: Glycosylated hemoglobin; FPG: Fasting Plasma Glucose; LDL-C: Low-Density Lipoprotein Cholesterol; TG: Triglycerides; CRP: C-reactive protein; Hb: Hemoglobin; ALB: Albumin; ALT: Alanine Aminotransferase; AST: Aspartate Aminotransferase.

Table S11 Comparison of AUC values between lipid metabolism-related inflammatory indices (LMIIs)

| Test | AUC | *P* value | Bonferroni-adjusted *P* | 95% CI | Optimal threshold | Specificity | Sensitivity | Youden index |
| --- | --- | --- | --- | --- | --- | --- | --- | --- |
| Base + PHR | 0.750 | 0.00041 | 0.00246 | 0.732 - 0.768 | 0.284 | 0.689 | 0.689 | 0.378 |
| Base + NHR | 0.759 | NA | NA | 0.741 - 0.776 | 0.302 | 0.729 | 0.661 | 0.390 |
| Base + MHR | 0.754 | 0.03667 | 0.22001 | 0.736 - 0.772 | 0.301 | 0.727 | 0.661 | 0.388 |
| Base + LHR | 0.750 | 0.00053 | 0.00320 | 0.731 - 0.768 | 0.282 | 0.686 | 0.694 | 0.380 |

The threshold represents the optimal predicted probability from the corresponding multivariable logistic regression model and is not a cutoff value of the LMII itself. *P* values were calculated for pairwise comparisons of AUCs between each LMII-based model and the NHR-based model. Bonferroni-adjusted *P* values were used to account for multiple comparisons.The base model includes age, sex, ethnicity, diabetes duration, FPG, HbA1c, CRP, Hb, ALB, ALT, AST, LDL-C, TG, hypertension, CVD, obesity, antidiabetic medications, lipid-lowering agents, aspirin, RAAS inhibitors, smoking and drinking.

**Abbreviations:** PHR: Platelet-to-high-density lipoprotein cholesterol ratio; NHR: neutrophil-to-high-density lipoprotein cholesterol ratio; MHR: monocyte-to-high-density lipoprotein cholesterol ratio; LHR: lymphocyte-to-high-density lipoprotein cholesterol ratio.

Table S12. Incremental predictive value of lipid metabolism-related inflammatory indices (LMIIs) beyond the base model for diabetic kidney disease (DKD)

| Models | Continuous NRI (p-value) | IDI (p-value) | ΔAUC (p-value) |
| --- | --- | --- | --- |
| Base + PHR | 0.111 (0.003) | 0.002 (0.014) | 0.003 (0.056) |
| Base + NHR | 0.276 (< 0.001) | 0.014 (< 0.001) | 0.011 (< 0.001) |
| Base + MHR | 0.176 (< 0.001) | 0.009 (< 0.001) | 0.007 (0.003) |
| Base + LHR | 0.102 (0.007) | 0.002 (0.017) | 0.002 (0.095) |

The base model includes age, sex, ethnicity, diabetes duration, FPG, HbA1c, CRP, Hb, ALB, ALT, AST, LDL-C, TG, hypertension, CVD, obesity, antidiabetic medications, lipid-lowering agents, aspirin, RAAS inhibitors, smoking and drinking.

**Abbreviations:** ΔAUC, change in the area under the receiver operating characteristic curve between the extended and core models; NRI, continuous net reclassification improvement; IDI, integrated discrimination improvement. PHR, platelet-to-high-density lipoprotein cholesterol ratio; NHR, neutrophil-to-high-density lipoprotein cholesterol ratio; MHR, monocyte-to-high-density lipoprotein cholesterol ratio; LHR, lymphocyte-to-high-density lipoprotein cholesterol ratio. ΔAUC, continuous NRI, and IDI were calculated for the core and LMII-extended models and pooled across five imputed datasets according to Rubin’s rules.
